# Comparison of genomic-enabled cross selection criteria for the improvement of inbred line breeding populations

**DOI:** 10.1093/g3journal/jkad195

**Published:** 2023-08-25

**Authors:** Alice Danguy des Déserts, Nicolas Durand, Bertrand Servin, Ellen Goudemand-Dugué, Jean-Marc Alliot, Daniel Ruiz, Gilles Charmet, Jean-Michel Elsen, Sophie Bouchet

**Affiliations:** INRAE-Université Clermont-Auvergne, UMR1095, GDEC, 63000 Clermont-Ferrand, Puy de Dôme, Auvergne, France; INRAE-Université de Toulouse, UMR1388, GenPhySE, 31320 Castanet-Tolosan, Haute-Garonne, Occitanie, France; ENAC-Ecole Nationale de l'Aviation Civile, 31000 Toulouse, Haute-Garonne, Occitanie, France; INRAE-Université de Toulouse, UMR1388, GenPhySE, 31320 Castanet-Tolosan, Haute-Garonne, Occitanie, France; Florimond-Desprez Veuve & Fils SAS, 59242 Cappelle-en-Pévèle, Nord, Hauts-de-France, France; IRIT-APO, Institut de recherche en informatique de Toulouse - Algorithmes Parallèles et Optimisation, 31000 Toulouse, Haute-Garonne, Occitanie, France; INPT-ENSEEIHT, Institut National Polytechnique de Toulouse, École Nationale Supérieure d'Électrotechnique, d'Électronique, d'Informatique, d'Hydraulique et des Télécommunications, 31000 Toulouse, Haute-Garonne, Occitanie, France; INRAE-Université Clermont-Auvergne, UMR1095, GDEC, 63000 Clermont-Ferrand, Puy de Dôme, Auvergne, France; INRAE-Université de Toulouse, UMR1388, GenPhySE, 31320 Castanet-Tolosan, Haute-Garonne, Occitanie, France; INRAE-Université Clermont-Auvergne, UMR1095, GDEC, 63000 Clermont-Ferrand, Puy de Dôme, Auvergne, France

**Keywords:** cross value, mating design, genetic gain, diversity management, bread wheat, Genomic Prediction, GenPred, Shared Data Resources

## Abstract

A crucial step in inbred plant breeding is the choice of mating design to derive high-performing inbred varieties while also maintaining a competitive breeding population to secure sufficient genetic gain in future generations. In practice, the mating design usually relies on crosses involving the best parental inbred lines to ensure high mean progeny performance. This excludes crosses involving lower performing but more complementary parents in terms of favorable alleles. We predicted the ability of crosses to produce putative outstanding progenies (high mean and high variance progeny distribution) using genomic prediction models. This study compared the benefits and drawbacks of 7 genomic cross selection criteria (CSC) in terms of genetic gain for 1 trait and genetic diversity in the next generation. Six CSC were already published, and we propose an improved CSC that can estimate the proportion of progeny above a threshold defined for the whole mating plan. We simulated mating designs optimized using different CSC. The 835 elite parents came from a real breeding program and were evaluated between 2000 and 2016. We applied constraints on parental contributions and genetic similarities between selected parents according to usual breeder practices. Our results showed that CSC based on progeny variance estimation increased the genetic value of superior progenies by up to 5% in the next generation compared to CSC based on the progeny mean estimation (i.e. parental genetic values) alone. It also increased the genetic gain (up to 4%) and/or maintained more genetic diversity at QTLs (up to 4% more genic variance when the marker effects were perfectly estimated).

**List of CSC**

**Table:** 

CSC	Definition for a given cross
PM	parental mean GEBV value = expected progeny mean value
UC1	expected mean value of the top 7% progeny
UC2	expected mean value of the top 0.01% progeny
UC3	expected mean value of progeny superior to the 93% quantile of the whole mating design
PROBA	expected percentage of progeny superior to a threshold, set to the best parental value in this study
EMBV	expected maximum haploid breeding value expected value of the best progeny among D progenies
OHV	Optimal haploid value best theoretical progeny value (taking the best allele at QTL)

## Introduction

Plant breeders have 2 main objectives—derive high-performing varieties at each cycle and improve the mean genetic value of their germplasm so as to be able to generate superior varieties in future generations. The mating design, i.e. the choice of the set of parental lines to cross and their combination, as well as the progeny size per cross, is critical to ensure both short- and long-term genetic gains. However, the number of candidate crosses is putatively very high while the number of crosses and progenies that can be experimentally tested is often limited.

Breeders can decide on the mating design by ranking crosses according to *cross selection criteria* (CSC) that estimate their ability to produce superior progenies for a given trait of interest. The simplest way to rank crosses is based on the expected mean genetic value of the progeny that can be estimated by the mean additive genetic value of the parental lines, the so-called parental mean (PM) criterion (Jinks and Pooni 1976). However, this criterion does not use information on the genetic variance of progeny derived from a cross (e.g. the progeny variance) and thus does not differentiate, among crosses of similar PM, those with a higher potential to generate extreme (transgressive) progenies, i.e. superior to the best parent, resulting in higher genetic gain. Several attempts have been made to predict the potential of a cross to produce high means but also extreme genetic variance in the progeny.

The progeny/gametic variance for inbreds/outbreds depends on the complementarity of favorable alleles between parents and their probability of recombining during meiosis (Zhong and Jannink 2007). Indeed, considering 2 QTLs, when alleles are in coupling phase (i.e. one parent carries the 2 beneficial alleles while the other carries deleterious ones), recombination decreases the progeny variance, while recombination increases this variance in repulsion phase. Regarding QTLs along the whole genome, progeny variance increases with the level of polymorphism between parents. In the past, genetic values were estimated via phenotypic observations [phenotypic selection (PS)]. Phenotypic and then genotypic distances were assumed to reflect parental genetic complementary and were used to predict cross progeny variance (Souza and Sorrells 1991; Bohn *et al.* 1999; Utz *et al.* 2001; Hung *et al.* 2012).

More recently, genomic prediction [genomic selection (GS)] was developed to estimate genetic values from genotypes [genomic estimated breeding value (GEBV)]. GS uses a training population (TP), which is phenotyped and genotyped to estimate the effects of segregating genomic variants (markers). Assuming that marker effects are additive, the GEBV of an individual is the sum of its allele effects at every marker. According to several simulation studies (Bernardo and Yu 2007; Bernardo 2009; Lorenzana and Bernardo 2009; Heffner *et al*. 2010; Heffner, Jannink, Iwata, *et al.* 2011; Heffner, Jannink and Sorrells 2011), compared to PS, GS can reduce the generation interval in crops via genotyping—rather than phenotyping—and rapid cycles. Depending on the species and the quality of the TP used to build the prediction model, GS can also increase the prediction accuracy (Lorenzana and Bernardo 2009; Heffner, Jannink, Iwata, *et al*. 2011; Heffner, Jannink and Sorrells 2011).

Genomic predictions offer a promising alternative to estimate progeny variance using marker effects and recombination rate estimates. The progeny distribution can be estimated by simulating progeny in silico (stochastic simulation), placing recombination of parental genomes along chromosome sequences according to a recombination map (Bernardo and Charcosset 2006; Mohammadi *et al*. 2015). Depending on progeny size, the probability to get outstanding lines may vary, which can be taken into account in stochastic simulations. But it is compute intensive. Alternatively, progeny distribution can be predicted using analytical formulas. To do so, the progeny breeding value distribution is assumed to be Gaussian, which is expected for traits controlled by a very high number of variants with small effects. The Gaussian distribution is centered on the expected progeny mean (progeny mean = PM), which can be estimated from the mean of additive parental genetic values using PS or GS. A formula to predict inbred progeny variance derived from a cross between 2 inbred lines was reported by Lehermeier *et al*. (2017) based on marker effect estimates using GS and their cosegregation in progeny derived from a genetic map, taking into consideration the type and generation of biparental population. Formulas were also derived to estimate 3- and 4-way cross progeny variance (Allier, Lehermeier, *et al*. 2019) and to predict gametic variance in an animal breeding context (Santos *et al*. 2019).

Several CSC using progeny distribution estimates have been put forward, with each having strengths and weaknesses. One strategy consists in estimating the genetic value of the best inbred progeny that could be derived from a cross. Daetwyler *et al*. (2015) defined the optimal haploid value (OHV) corresponding to the genetic value of the progeny of a cross that would cumulate the most desirable alleles or haplotypes of parents at each position. OHV is fast to implement, and the selection of crosses based on this value has been shown to increase both the genetic values and genetic diversity of the superior fraction of progeny at the next generation, as compared to progeny derived from PM-based selection of crosses (Daetwyler *et al*. 2015; Lehermeier *et al*. 2017). Note that there is a very low probability of observing OHV in progeny as a high number of beneficial recombination events would be needed while avoiding all disadvantageous ones. Considering that the progeny size is generally limited, another CSC named expected maximum haploid breeding value (EMBV) was suggested by Müller *et al*. (2018). EMBV predicts the value of a cross as the expected mean of the *K* top progenies among *D* allocated to the cross.

Another strategy is to predict the average genetic value of a superior fraction of the progeny of candidate crosses. Schnell and Utz (1975) suggested ranking crosses based on the expected mean of an upper fraction *q* of their progeny. This CSC was named the usefulness criterion (UC), with UC = PM + i*h*σ, where *i* is the selection intensity corresponding to the fraction *q* of selected progenies, *h* is the square root of heritability, and *σ* is the progeny variance in our context. Note that when using UC in a GS context, *h*^2^ (and thus *h*) is usually set at 1 for GEBV, but further research would be required to be sure that this assumption has no influence on the results. As an alternative to UC, Wellmann (2019) and Bijma *et al*. (2020) suggested computing the value of a cross as the probability of producing progeny superior to a given threshold. This threshold can be extrapolated from historical genetic gains observed in the breeding program (Wellmann 2019), or it can be estimated as corresponding to the usual per-generation selection rate among progeny (Bijma *et al*. 2020). It can also simply be the genetic value of the best parental line.

Several studies compared the efficiency of those CSC in short-term selection responses (one generation) (Zhong and Jannink 2007; Lehermeier *et al*. 2017; Yao *et al*. 2018; Bijma *et al*. 2020). The findings showed that CSC based on progeny variance estimation could actually increase the genetic gain, even if the parental genetic values and progeny variance were not accurately estimated. Zhong and Jannink (2007) and Bijma *et al*. (2020) showed that the relative benefits of CSC based on progeny variance estimation compared to PM depend on the ratio between the variance of progeny SD—var(*σ*)—and the variance of progeny means—var(PM)—in the list of candidate crosses. When var(PM) among crosses is highly superior to var(*σ*), PM alone is enough to predict the rank of crosses.

According to the breeder's equation, genetic gain is proportional to the genetic diversity and selection intensity (Falconer and Mackay 1996). In a closed breeding program, i.e. with no external genitors involved, the diversity decreases as the selection efficiency increases. A further objective of the mating design is thus to maintain sufficient genetic diversity to ensure long-term genetic gain. Breeders empirically avoid crossing the most related genitors (Wartha and Lorenz 2021) while ensuring that a sufficient number of parental lines will contribute to the next generation. Several more advanced methods have been designed to balance the expected genetic gain and expected genetic diversity at successive generations when selecting genitors and/or crosses, e.g. by constraining the average genetic similarity of all selected parents (Toro and Perez-Enciso 1990; Meuwissen 1997; Jannink *et al*. 2010; Akdemir *et al*. 2019; Allier, Lehermeier, *et al*. 2019). In any case, the sought after balance between the expected genetic gain and expected genetic diversity is not trivial to define. It depends on whether the objective is to optimize short- or long-term genetic gain (e.g. in a breeding or prebreeding program).

We tested here the hypothesis that it is useful to estimate the variance in the progeny to optimize the mating design in order to increase short-term genetic gain and diversity instead of using PM information only, in a French winter bread wheat breeding program. We compared the genetic values and genetic diversity of top inbred progenies derived from optimized mating designs obtained using different CSC. The parental population included 835 historical (2000–2016) lines from the French National Research Institute for Agriculture, Food and Environment (INRAE)-Agri-Obtentions (AO) winter bread wheat breeding program. We tested several previously published CSC (PM, OHV, EMBV, and UC) and adapted 2 new ones from the literature that had never been tested per se. From Wellmann (2019), we adapted the probability of a given cross progeny to exceed a given threshold (the best parental value in this study) (PROBA), which consisted of ranking crosses based on the expected proportion of progeny superior to the best breeding line of the breeding program. From Bijma *et al*. (2020), we defined the UC3 criterion maximizing the expected value of a superior fraction of the whole progeny of the mating design, without any approximation or hypothesis. We compared genetic gain and diversity levels in the selected progeny when the QTL effects and positions were supposedly known and also when the marker effects were estimated using a GBLUP model with observed parental phenotypes. Diversity constraints on parental contributions, i.e. minimal and maximal number of parents, crosses, and progenies, were chosen according to common breeding practices.

## Materials and methods

### Parental populations

The founder population included 835 F_8_–F_9_ winter-type bread wheat lines developed and phenotyped between 2000 and 2016 by breeders from the INRAE and its subsidiary breeding company AO (Ben Sadoun *et al*. 2020). They were genotyped with 35k SNPs (Ben Sadoun *et al*. 2020) representative of the TaBW280K array (Rimbert *et al*. 2018). For this analysis, the markers were filtered according to the missing data rate (<5%), heterozygosity rate (<5%), and minor allele frequency (>10%) yielding 16,429 SNPs. Missing genotypes were imputed using the Beagle v4.1 algorithm (Browning and Browning 2007, 2016) implemented in the synbreed R-package (Wimmer *et al*. 2012). The genetic values for yield of these 835 lines were estimated using the GBLUP model.

### Different tested scenarios

**Fig. 1.**
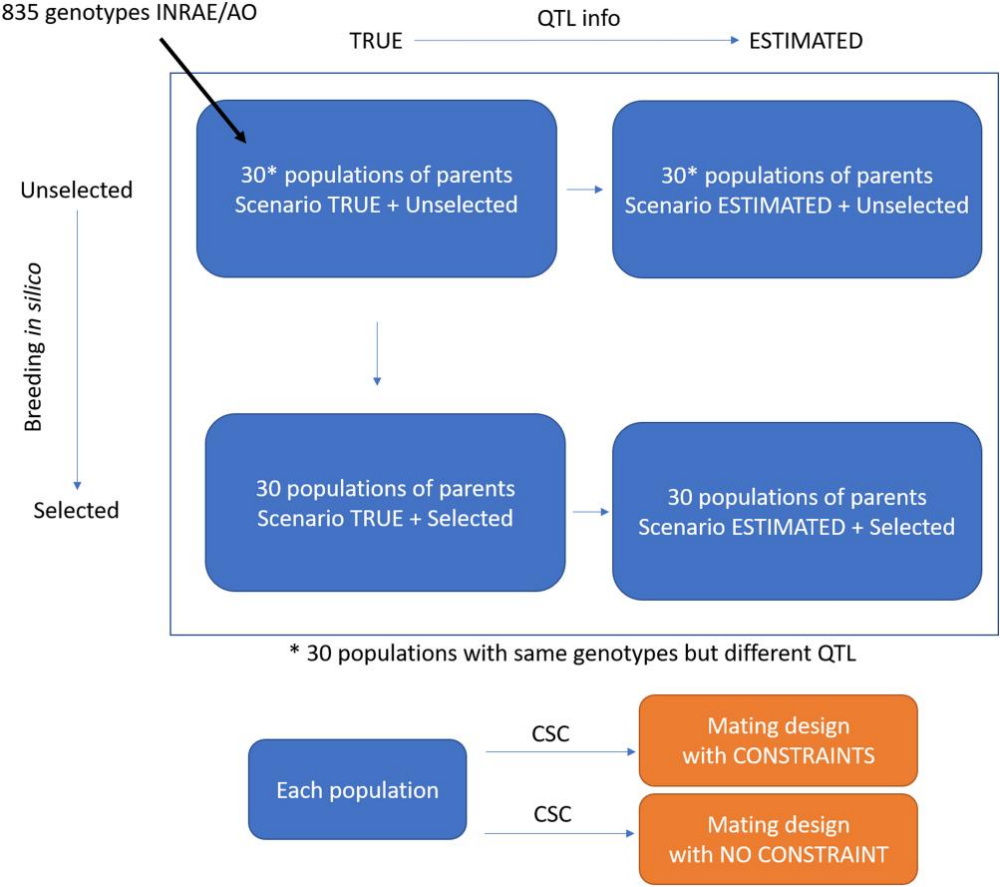
Different tested scenarios. The scenarios considered 2 marker effect estimation accuracy levels (TRUE, in which QTL effects were known, and ESTIMATED, with marker effects being estimated by GS); 2 types of populations (Unselected populations corresponding to the 835 INRAE-AO founders and Selected populations starting from those founders, followed by 3 random crossing and selection cycles); and 2 mating design constraint levels (CONSTRAINT and NO CONSTRAINT). Each scenario was simulated for 30 different genetic architectures (characterized by a set of 300 QTLs with random position and effect) using INRAE-AO historical breeding lines as the parental population.

Simulations were carried out to take 3 parameters into account:

1. The degree of selection for the trait of interest in the parental population.
  (1a) Unselected population: we considered that the parental population composed of 835 historical breeding lines from INRAE-AO had never been selected for the trait of interest. QTL positions and effects were randomly assigned.
  (1b) Selected population: an ancestral population created as in (1a) was further crossed and selected via 3 in silico cycles to produce the parental population. Note that the genetic architecture of this population was the same as the corresponding Unselected parental population, i.e. QTL and their effects were simulated in the starting Unselected population.
2. The accuracy of marker effect estimates.
  (2a) *TRUE*: QTL effects and positions were supposedly known (or perfectly estimated). The TRUE scenario provides information about the maximum potential of CSC if the TP is optimal and marker effects are perfectly estimated. If the relative performances of CSC in this ideal scenario are not convincing, there is no use implementing them in breeding programs.
  (2b) *ESTIMATED*: marker effects were estimated by GS using parental simulated phenotypes and removing QTLs in genotypes (Fig. 1). We tested the same 30 different trait architectures for each scenario.
3. The constraints to maintain genetic diversity in the breeding material.
  (3a) *CONSTRAINT* on parental contributions and genetic distance between parents (see below, constraints C1–C6).
  (3b) *NO CONSTRAINT* (only constraints C1 and C2 were applied to the total number of progenies and the minimum and maximum number of progenies per cross).

We simulated the 8 scenarios that are summarized in Fig. 1. Note that the corresponding CONSTRAINT/NO CONSTRAINT and TRUE/ESTIMATED scenarios were simulated with the same parental population and genetic architecture.

### Unselected population + TRUE QTL effect scenario

The parental population was built with genotypes of the 835 historical breeding lines from the INRAE-AO breeding program. In order to take into account the uncertainty in the genetic determinism of quantitative traits, we simulated 30 random genetic architectures controlled by 300 QTLs randomly picked among the 16k SNPs, with normally distributed genetic effects N(0,1). The favorable allele was assigned at random to 1 of the 2 SNP alleles so that coupling and repulsion associations would also occur at random. As progeny genetic variance is related to parental variance (Mrode 2005), historical yield data of the TP were used to estimate a realistic genetic variance. QTL effects were adjusted to provide a variance of true breeding values (TBV) of 14 (quintal/ha)^2^. TBV were calculated as the cross product between QTL effects and allelic doses.

### Selected population + TRUE QTL effect scenario

Populations under selection for 1 or several traits of interest present negative covariances between QTLs. This phenomenon is called the Bulmer effect (Bulmer 1971). Hence, the observed genetic variance is lower compared to populations that have never been under selection. In unselected population simulations, this phenomenon was not taken into account as QTLs and effects were assigned at random positions along the genome. To take the Bulmer effect into account, we derived 30 “selected populations” from the founders by applying 3 truncation selection cycles to the 30 Unselected populations. At each of the 3 selection cycles, 300 crosses were performed at random from the 300 lines with the highest TBV. Selection on TBV provided an opportunity to maximize the Bulmer effect in new populations. Each cross produced 11 F5 Recombinant Inbred Lines (RIL) (total progeny = 3,300), simulated with the MOBPS R package (Pook *et al*. 2020). At cycles 1 and 2, only 1 progeny per cross was selected based on TBV. In the 3rd cycle, the 3 best progenies per cross were kept, leading to a final population of 900 parental lines and called the “selected population,” from which 835 lines were sampled at random.

### Unselected population + ESTIMATED QTL effect scenario

Phenotypes of unselected parents were simulated with a heritability $h_{0}^{2}$ of 0.4 by adding a normally distributed noise of variance 21 (quintal/ha)^2^ to their TBV $\left(h_{0}^{2}=14/(14+21)=0.4\right)$.

Marker effects were estimated by backsolving the model using the PostGSf90 software package (Wang *et al*. 2012; Aguilar *et al*. 2014). GEBV of progenies were computed as the cross product between estimated marker effects and allelic doses.

### Selected population + ESTIMATED QTL effect scenario

Phenotypes were simulated by adding a normally distributed noise of variance 21 (quintal/ha)^2^ to the TBV. We used the same procedure as above to estimate marker effects and GEBV.

### Estimation of genetic values and marker effects

For the ESTIMATED scenarios, we used a GBLUP model to estimate parental line genetic values and marker effects according to the following model:


$$Y_{i}=\mu +\alpha_{i}+e_{i},$$

where *i* denotes the name of the parental line (*n* = 835), *Y* is the vector of phenotypes, *μ* is the average phenotype, *α* is the vector of genetic values, and *e* is the vector of residual effects. The genetic values were assumed to follow $N(0,G^{\left(1\right)}\sigma_{a}^{2})$, where *G*^(1)^ is the genomic relationship matrix computed as $ZZ^{\prime }/2\sum_{l}\;p_{l}(1-p_{l})$, with *Z* being the centered genotyping matrix, excluding QTL genotype, and $p_{l}$ the allelic frequency at locus *l*, and where $\sigma_{a}^{2}$ is the genetic variance. Residual effects were assumed to follow $N(0,\;I\sigma_{e}^{2})$. Parameters $\sigma_{a}^{2}$ and $\sigma_{e}^{2}$ were estimated using the AIREMLf90 software package (Misztal 2008).

### Prediction of progeny variance

The expected variance of progeny was computed using the formula provided by Lehermeier *et al*. (2017) for biparental RIL progeny obtained after 4 generations of selfing (F5 RILs). For each cross $P_{i}*P_{j}$, the formula for the expected variance of progeny was as follows:


$$\begin{array}{rl} \sigma_{\;\;i,j}^{2^{RILs\;F5}} & =4\;*\;(\sum_{l=1}^{L}\beta_{l}^{2}p_{l\;ij}(1-p_{l\;ij})+2\sum_{k<l}\beta_{k}\beta_{l}4D_{kl\;ij}(1-2r_{{kl}^{5}}\\ & \;\;\;\;-(0.5(1-2r_{kl}){)}^{5})), \end{array}$$

where *β* is either QTL effects for TRUE scenarios (length *β* = 300) or estimated marker effects for ESTIMATED scenarios (length *β* = 16,429–300); $p_{l\;ij}$ is the allelic frequency at locus *l* for parents $P_{i}$ and $P_{j}$ (0 if parents carry the same allele at this locus, 0.5 if they differ); $D_{kl\;ij}$ is the linkage disequilibrium (LD) between alleles at loci *l* and *k* for parents $P_{i}$ and $P_{j}$ [either 0 if parents carry the same allele at locus *l* or *k* or 0.25 if alleles are in coupling phase (i.e. 1 parent carries the 2 beneficial alleles while the other carries deleterious alleles) or 0.25 if the alleles are in repulsion phase]; and $r_{kl}$ is the recombination rate between locus *l* and *k*. The recombination rates were computed from the Western European recombination map published by Danguy des Déserts *et al*. (2021), using the Haldane mapping function (Haldane and Waddington 1931): $r_{kl}=0.5*(1-e^{-2d_{kl}})$, where $d_{kl}$ is the genetic distance [in morgans (M)] between loci *k* and *l* (Haldane 1919).

The estimation of progeny variance for a high number of crosses (348,195 crosses in our study) and of simulations (*n* = 120) was highly time consuming. We accelerated this estimation as described in Supplementary Protocol 1.

### Mating design constraints

Selecting crosses with the best CSC while including constraints on the progeny allocation across parents can be defined as an optimization problem in which variables to adjust (progeny sizes of each candidate cross in our case) will determine the value of the objective function to maximize (the sum of products of CSC values by progeny sizes in our case) but are also subject to constraints (e.g. the number of progeny per cross and per parent could be limited). When the equation system is linear for the variables to adjust, linear programming may be used to find the set(s) of variables that maximize the objective function. Otherwise, for more complex problems, heuristic algorithms such as genetic algorithms (GAs) may be used to obtain a good (but not necessarily the best) problem solution.

A mating design was defined by a vector giving the number of progenies $D_{ij}\;$ allocated to each candidate cross $P_{i}*P_{j}$. The constraints were inspired from the bread wheat breeding program of the private company Florimond Desprez (personal communication):

- C1: the total number of progenies was set at *D* = 3,300.
- C2: the number of progenies allocated to a cross ranged from $D_{\min}=5\;\mathrm{to}\;D_{\max}=60.$
- C3: the number of crosses ranged from $K_{\min}=200\mathrm{to}\;K_{\max}=300.$
- C4: the number of progenies derived from 1 parent could not exceed $C_{\max}=250.$
- C5: the number of recruited parents for the mating design ranged from $P_{\min}=100\;\mathrm{to}\;P_{\max}=132.$
- C6: highly related parental lines could not be crossed. We used the LDAK software package (Speed *et al*. 2012) to obtain a genomic relationship matrix *G*^(2)^ in which SNPs were weighted according to local LD in order to take into account the very heterogenous LD in bread wheat, which markedly increases from telomeres to centromeres. This variance–covariance matrix was computed as WW’, where *W* was obtained by centering and scaling each column of the genotyping matrix *Z* such that $\;W_{l}=\;w_{l}\;*(Z_{l}-p_{l})/\sqrt{\;p_{l}(1-p_{l})},$ where $p_{i}$ is the allelic frequency at locus *l* and $w_{l}$ is the weight estimated by LDAK according to the local LD intensity. Crosses involving a pair of parental lines showing covariance superior to the 99% quantile covariance value were removed from the list of candidate crosses (1% of the candidate crosses).

We compared scenarios with and without constraints, i.e. respectively called “CONSTRAINT” and “NO CONSTRAINT.” Only constraints C1 and C2 were considered for the NO CONSTRAINT scenarios. Note that parental lines GEBV and estimates of marker effects were the same for the CONSTRAINT and NO CONSTRAINT scenarios.

In summary, we compared the benefits of CSC for 8 scenarios: 2 scenarios that differentiated the type of parental population (Unselected or Selected), 2 scenarios with different genomic prediction accuracies (TRUE or ESTIMATED), and 2 scenarios with different diversity constraints applied on the mating designs (CONSTRAINT and NO CONSTRAINT).

### CSC and their corresponding objective function

One mating design is defined by a set of crosses and their respective number of progenies. For each CSC, the mating design maximizes a specific objective function under constraints C1–C6 for the CONSTRAINT scenarios and C1 and C2 for the NO CONSTRAINT scenarios.

#### PM

The usefulness of the $P_{i}*P_{j}$ cross is the expected progeny mean, estimated as follows:


$$PM_{ij}=\frac{\alpha_{i}+\alpha_{j}}{2},$$

where *α* is either the TBV of parents for TRUE scenarios or GEBV for ESTIMATED scenarios. The objective function to maximize is the following:


$$\underset{i,j}{\sum }\;D_{ij}\;*\;PM_{ij}.$$

#### UC1

This CSC is the expected mean of the *q* = 7% best progeny of a cross, computed as follows (Schnell and Utz 1975):


$$\mathrm{UC}1_{ij}=PM_{ij}+i^{q=7\text{\%}}*\sigma_{ij},$$

where $i^{q=7\text{\%}}$ ∼ 1.91 is the selection intensity corresponding to a 7% selection rate (computed as the inverse Mills ratio) and $\sigma_{ij}$ is the progeny SD. The progeny SD $\sigma_{ij}$ is computed either with QTL effects for TRUE scenarios or estimated allelic effects for ESTIMATED scenarios. Note that a 7% selection rate is usually applied at the Florimond Desprez company between F5 and F6 generations (when genomic predictions are applied). The objective function to maximize is the following:


$$\underset{i,j}{\sum }\;D_{ij}\;*\;\mathrm{UC}1_{ij}.$$

#### UC2

This CSC is the expected mean of the *q* = 0.01% best progeny of a cross, computed as follows:


$$\mathrm{UC}2_{ij}=PM_{ij}+i^{q=0.01\text{\%}}*\sigma_{ij},$$

where $i^{q=0.01\text{\%}}$ ∼ 4 is the selection intensity corresponding to a 0.01% selection rate, i.e. twice the selection intensity of the UC1 criterion. Although this 0.01% selection rate is not realistic considering the small progeny size ($D_{\max}$ = 60 progenies per cross), the objective is to select crosses with higher expected genetic variance compared to the UC1 criterion while counting on them providing more outstanding progenies. The corresponding objective function to maximize is the following:


$$\underset{i,j}{\sum }\;D_{ij}\;*\;\mathrm{UC}2_{ij}.$$

#### EMBV

The expected value of the best progeny among $D_{ij}$ allocated to a cross is the following (Müller *et al*. 2018):


$$\mathrm{EMB}V_{ij}(D_{ij})\;=PM_{ij}+\mathrm{IN}T^{1/D_{ij}\;}*\sigma_{ij},$$

with $\mathrm{IN}T^{1/D_{ij}\;}$ being the expected value of the highest order statistic among a sample of $D_{ij}$ statistics drawn from *N*(0,1). An approximation of $\mathrm{IN}T^{1/D_{ij}\;}$ was provided by the following (Burrows 1972):


$$\mathrm{IN}T^{N/M\;}=i^{q=\;N/M\;}-\frac{(M-N)*q}{2N(M+1)*f(y_{q})},$$

where *f* is the density function of a Gaussian law *N*(0,1) and $y_{q}$ is the truncation threshold such that $P(y\;\geq \;y_{q})=q=N/M$. In our conditions, *N* = 1, *M* = $D_{ij}$, and $i^{q\;=N/M}=f(y_{q})/q$, so the formula of Burrows yields the following:


$$\mathrm{IN}T^{1/D_{ij}\;}=\;i^{q_{ij}=1/D_{ij}\;}-\frac{D_{ij}-1}{2*(1+D_{ij})*i^{q_{ij}=1/D_{ij}\;}}.$$

The objective function to maximize is the following:


$$\underset{i,j}{\sum }\;D_{ij}\;*\;\mathrm{EMB}V_{ij}(D_{ij}).$$

#### PROBA

This criterion ranks crosses based on their ability to produce a progeny exceeding a threshold *λ*, as suggested by Wellmann (2019) and Bijma *et al*. (2020). For setting *λ*, we use the genetic value (TBV for TRUE scenarios or GEBV for ESTIMATED scenarios) of the best parental line. The probability of a $P_{i}*P_{j}$ cross producing progeny with a genetic value superior to *λ* is $q_{ij}^{\lambda }=1-F_{ij}(x\leq \lambda )$, with $F_{ij}$ the cumulative distribution function of the Gaussian distribution $N(PM_{ij},\;\sigma_{ij}^{2})$. The probability that no progeny of the $P_{i}*P_{j}\;$ cross exceeds *λ* is $(1-q_{ij}^{\lambda }{)}^{D_{ij}}$. The probability that no progeny from all crosses exceed *λ* is $\prod_{i,j}{\left(1-q_{ij}^{\lambda }\right)}^{D_{ij}}$, so the log probability is $\sum_{i,j}D_{ij}*\log(1-q_{ij}^{\lambda })$. Maximizing the probability that at least 1 offspring will have genetic value greater than *λ* is equivalent to minimizing the objective function:


$$\underset{i,j}{\sum }\;D_{ij}\;*\;\log\;(1-q_{ij}^{\lambda }).$$

#### UC3

This criterion aims to maximize the expected mean of the superior quantile *q* (e.g. *q* = 7%) of progenies of the whole mating design, where *q* is the usual proportion of selected progenies. The same selection threshold $s_{q}$ is applied to all crosses and corresponds to the superior quantile *q* of the progeny genetic value distribution. The expected proportion of progeny of genetic value superior to $s_{q}$ differs for each cross, and the total proportion of progeny exceeding $s_{q}$ is equal to *q*:

$q=\left(\sum_{i,j}D_{ij}*q_{ij}^{s_{q}}\right)/D$ , where $q_{ij}^{s_{q}}=\;1-\;F_{ij}(x\;\leq \;s_{q})$ is the expected proportion of progeny superior to $s_{q}$ within the $P_{i}*P_{j}$ family. The expected value of progeny superior to $s_{q}$ within each family is equal to $\mathrm{UC}3_{ij}^{s_{q}}=PM_{ij}+i^{q_{ij}^{s_{q}}\;}*\sigma_{ij}$. For a given mating design, as defined by the vector of $D_{ij}$, the expected value of the *q* best progenies is thus equal to the following:


$$\underset{i,j}{\sum }\frac{D_{ij}\;*\;q_{ij}^{s_{q}}*\;\mathrm{UC}3_{ij}^{s_{q}}}{q\;*\;D}.$$

The best mating design is obtained by maximizing this objective function, with the constraint $D=\sum_{i,j}D_{ij}\;*\;q_{ij}^{s_{q}}$.

#### OHV

Daetwyler *et al*. (2015) defined OHV as the value of the best inbred progeny that could be theoretically derived from a cross. For each genomic segment *b*, the effects of haplotypes carried by parents $P_{i}$ and $P_{j}$ are respectively called $\beta_{bi}$ and $\beta_{bj}$. The OHV of a cross is computed as follows:


$$\mathrm{OH}V_{ij}=\;2\;*\sum_{b}\max(\beta_{bi},\beta_{bj}).$$

Daetwyler *et al*. (2015) showed that selecting crosses based on OHV instead of PM was advantageous in terms of short-term genetic gain when the number of haplotypic blocks per chromosome was low. For bread wheat, they showed, by simulation, that 1–3 blocks per chromosome allowed higher genetic gain than smaller blocks. We defined 3 haplotypic blocks per chromosome, 1 block per chromosome arm, and 1 block for the centromere (with the positions of centromeric regions defined in Choulet *et al.* 2014).

The objective function to maximize is the following:


$$\underset{i,j}{\sum }\;D_{ij}\;*\;\mathrm{OH}V_{ij}.$$

### Optimization of mating designs

In the CONSTRAINT scenarios, for all CSC but EMBV and UC3, the objective function and constraints constituted a system of linear equations. We used an integer linear programming algorithm implemented in IBM ILOG CPLEX software (CPLEX Python API, IBM 2017) to maximize (or minimize) objective functions while respecting the constraints.

For criteria EMBV and UC3, the objective function and constraints did not form a system of linear equations, as the usefulness (e.g. the CSC value) of a cross actually depended on the number of progenies allocated to the cross. To optimize mating designs for EMBV and UC3 criteria, we used a GA. GAs are population-based metaheuristics inspired by Darwinism (Goldberg 1989). The GA description used in this study and the tuning parameters are given in Supplementary Protocol 2. GAs are difficult to tune and often remain stuck at local minima. To avoid premature convergence, a sharing process can be added before selection (Yin and Germay 1993) in order to give more chance to candidates that are isolated in the search space. The sharing process requires the definition of a distance between candidate solutions. Candidate solutions were considered different if at least 1 $D_{ij}$ was different. The population of candidate solutions per iteration was set at 100. At the first GA iteration, half of the initial candidate solutions were drawn at random, and the other half was set at linear programming optimization outputs of another CSC. The findings of a short preliminary study actually suggested that linear programming outputs of UC1 for EMBV optimization and PROBA outputs for UC3 optimization were the best starting points for EMBV and UC3 optimization.

For all criteria, we tested whether the preselection of candidate crosses with the highest PM would influence the value of the objective function to be maximized (Supplementary Table 1). For all criteria, preselection of the 10% highest PM crosses usually provided an objective function value after optimization that was 99% similar to the objective function value of the same population without preselection. To reduce the computation time, we thus optimized the mating designs with the 10% highest PM crosses. Note that preselection of crosses based on parental genetic values was also used in Zhong and Jannink (2007), Lehermeier *et al*. (2017), and Bijma *et al*. (2020).

In the NO CONSTRAINT scenarios, we did not use optimization software to optimize mating designs, except for UC3. For all other CSC, crosses were ranked based on CSC values, and the 55 best crosses received $D_{\max}$ = 60 progenies (constraint C2), for a total of *D* = 3,300 progenies (constraint C1).

### Progeny simulation

The F5 RIL progenies of each mating design were simulated using the MOBPS R package (Pook *et al*. 2020). Each mating design was simulated 20 times to account for the possibility that progeny genotypes might vary due to Mendelian gamete sampling. Progeny TBV were then computed as the cross product between QTL effects and the allelic dosage at QTL loci.

### CSC performance

The mating design optimization in this study had 2 objectives: to derive high-performing genotypes for commercial purposes and to improve the breeding population while limiting the loss of genetic diversity.

The ability of CSC to improve genetic values of commercial lines compared to PM was computed as the additional increase in the mean progeny TBV due to CSC compared to the increase due to PM (Bijma *et al*. 2020): $\frac{1}{\mathrm{PM}}\sum_{\;p*m}\frac{\mathrm{TB}V_{\;\mathrm{CSC}}\text{-}\mathrm{TB}V_{\;\mathrm{PM}}}{\mathrm{TB}V_{\;\mathrm{PM}}\text{-}\mathrm{TB}V_{\;\mathrm{parents}}}$,

where $\mathrm{TB}V_{\mathrm{CSC}}$ is the mean TBV of the *K* best progenies among *D* simulated progenies in the *m*th simulation (*M* = 20 repetitions) of a mating design optimized using CSC for the genetic architecture *p* (*P* = 30 different genetic architectures) for scenario *s*. The progeny selection rate (*K*/*D*) ranged from 1/3,300 (the very best progeny) to 10%. The term TBV stands for the mean TBV of all candidate parents.

Genetic diversity within progenies depends on the diversity of the selected parents and the progeny distribution across the selected parents and crosses.

To compare mating designs optimized according different CSC in terms of diversity management, we used 2 statistics, 1 considering the whole genome that calculates the genetic similarity between recruited parents (Supplementary Protocol 3) and 1 considering only the useful diversity for the considered trait at QTL, the so-called genic variance. It was computed as $\sigma_{genic\;p}^{2}=\sum_{l}4*\beta_{l\;p}^{2}*p_{l\;p}*(1-p_{l\;p})$, with $p_{l\;p}$ being the allelic frequency of QTLs in the selected progenies derived from population *p* in scenario *s* and $\beta_{l\;p}$ being the true allelic effect of QTLs at locus *l* in population *p* (note that QTL effect β did not change between scenarios, only between genetic architectures). The relative change in genic variance in the *K*/*D* selected progeny obtained using a mating design optimized for CSC compared to PM in scenario *s* was calculated as follows:


$$\frac{1}{\text{PM}}\sum_{p*m}\frac{\sigma_{\text{genic}}^{2}{\;}_{\text{CSC}}-\sigma_{\text{genic}}^{2}{\;}_{\text{PM}}}{\sigma_{\text{genic}}^{2}{\;}_{\text{PM}}-\sigma_{\text{genic}}^{2}{\;}_{\text{parents}}},$$

where $\sigma_{\mathrm{genic}\;\mathrm{CSC}}^{2}$ was the genic variance in the selected set of progenies in the *m*th progeny simulation for architecture *p*. To evaluate the ability of CSC to improve the new breeding population in terms of both gain and diversity, we set a 7% selection rate, corresponding to a realistic selection rate at the F5 stage in a bread wheat breeding program, and computed the relative increase in the mean progeny TBV and the relative increase in the progeny genic variance.

## Results

### Genetic gain in selected progeny

Crosses were selected using 7 genomic CSC, namely PM (parental mean GEBV value = expected progeny mean value), UC1 (expected mean value of the top 7% progeny), UC2 (expected mean value of the top 0.01% progeny), UC3 (expected mean value of progeny superior to the 93% quantile of the whole mating design), PROBA (expected percentage of progeny superior to a threshold, set to the best parental value in this study), EMBV (expected value of the best progeny among *D* progenies), and OHV [best theoretical progeny value (taking the best allele at QTL)]. They were computed with TRUE or ESTIMATED marker effects and using parents from Unselected or Selected populations for 30 different trait architectures.

We considered that the new breeding population included the 7% best progeny derived from the optimized mating design.

**Fig. 2.**
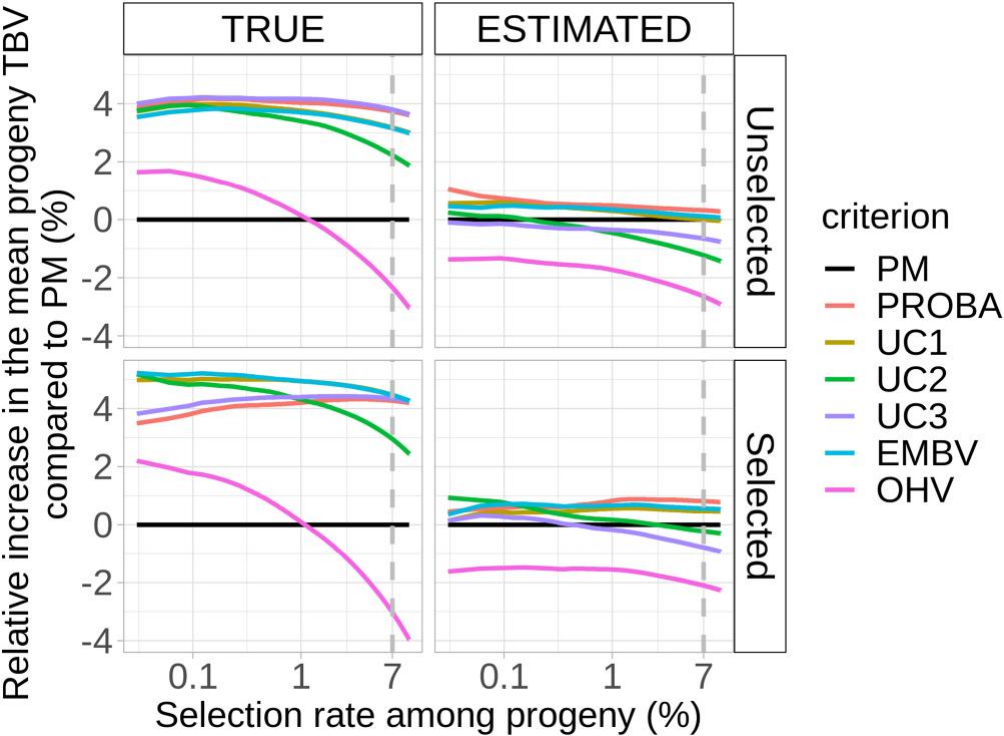
Relative increase in the 7% best progeny TBV using CSC instead of PM for CONSTRAINT scenarios. The vertical dashed line represents a 7% selection rate, as used in Fig. 3.

Figure 2 gives the additional increase in the mean progeny TBV due to mating optimization using CSC compared to the increase due to PM for a selection rate ranging from 0.03% (selection of the best progeny among *D* = 3,300 progenies) to 10%.

For TRUE scenarios, all criteria were superior to PM when selection was strong (selection rate < 1%). For example, for all CSC but OHV, the mean TBV of the selected progeny increased by around 4% for Unselected scenarios and by up to 5% for Selected scenarios compared to PM. For ESTIMATED scenarios, the relative increase barely exceeded 1% for all scenarios.

The ranking of criteria to maximize the TBV of selected progeny changed slightly with the scenario and selection rate. For TRUE + Unselected scenarios, the best criterion to maximize the value of the best progeny was UC3, with a 4.1% average increase in the TBV of the best progeny and a 1.9% SD; for TRUE + Selected scenarios, the best criterion was EMBV (5.2% ± 1.7%); for ESTIMATED + Unselected scenarios, the best criterion was PROBA (1.1% ± 3.1%); and for ESTIMATED + Selected scenarios, the best criterion was UC2 (0.9% ± 2.9%). Pairwise *t*-tests computed within each of the 4 scenarios identified 3 significant groups (*P* < 5% after Bonferroni correction) for TRUE scenarios: the upper group consisted of UC1, UC2, UC3, PROBA, and EMBV; the middle group consisted of PM; and the lower group consisted of OHV. The pairwise *t*-tests were not significant for the ESTIMATED scenarios, except for OHV, which was significantly lower than the other CSC. In conclusion, CSC alternatives to PM (except OHV) were superior to PM only for TRUE scenarios, with no substantial differences between them.

Note that PROBA and UC3 slightly underperformed for TRUE + Selected scenarios when selection was strong (low selection rate). Other CSC such as UC1 or UC2 should be preferred in that case. For all scenarios, the OHV criterion provided the lowest genetic gain. It was very disadvantageous compared to PM for all scenarios at >1% selection rate.

In conclusion, when QTL effects are perfectly estimated (TRUE scenarios), CSC based on progeny variance estimation (UC1, UC2, UC3, EMBV, and PROBA) could increase the genetic gain by up to 5% in breeding programs.

### Trade-off between genetic gain and genetic diversity in selected progeny

**Fig. 3.**
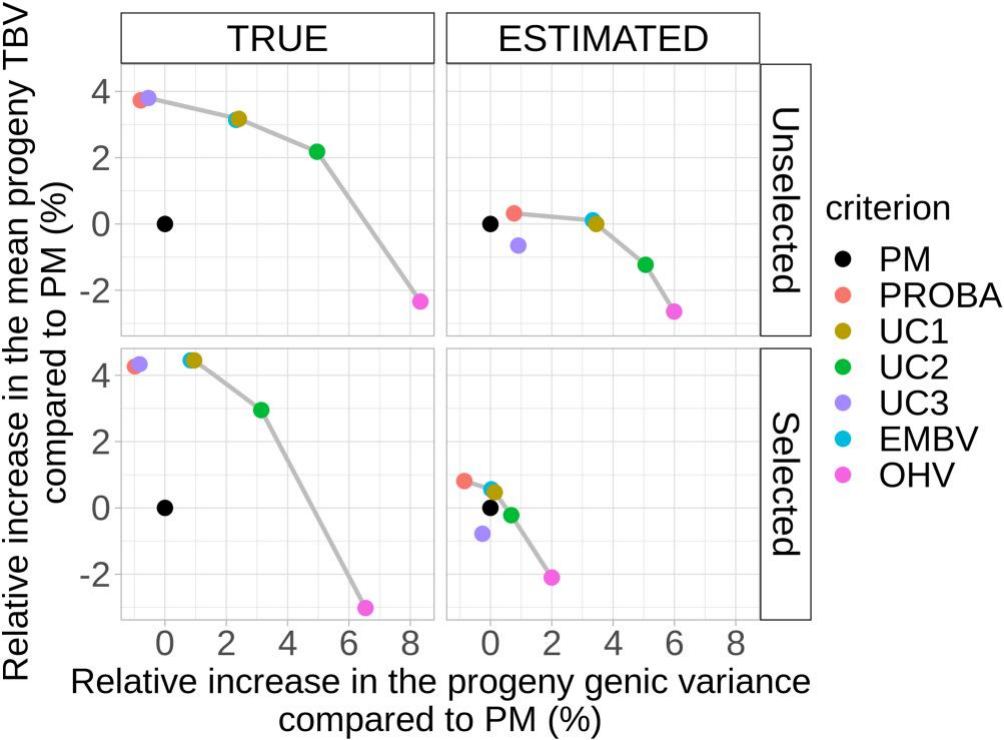
Trade-off between the relative increase in the 7% best progeny TBV and genic variance using CSC instead of PM for CONSTRAINT scenarios. Gray lines link criteria belonging to the set of best trade-offs, i.e. the best relative increase in the mean TBV for each level of relative increase in genic variance.

Figure 3 shows the trade-off between the relative increase in the 7% best progeny TBV and genic variance using CSC instead of PM. The gray line (pareto front) shows criteria with the best trade-off between genetic gain and genic diversity. For all scenarios (TRUE/ESTIMATED; Unselected/Selected), PM was not among the best trade-offs. In other words, all criteria are superior to PM in all scenarios, either in terms of genetic gain or diversity or both.

For example, for TRUE + Selected scenarios (bottom left in Fig. 3), crosses could be selected based on EMBV (blue point), UC1 (yellow point), or UC2 (green point), with these 3 CSC reducing the loss of genic variance up to 4% range compared to PM (black point). In fact, most CSC maintained more genic diversity than the PM criterion, except PROBA and UC3 for most scenarios.

The set of criteria providing the best trade-offs was similar for all scenarios and included OHV, UC2, UC1, and sometimes PROBA. There was a negative relationship between genetic gain and genetic diversity. For example, OHV was the most efficient criterion to maintain genetic diversity but the worst to maximize genetic gain, while PROBA was the opposite. UC1 and EMBV are better than PM in terms of genetic gain and slightly better in terms of diversity. UC2 is always better in terms of diversity and at least equivalent in terms of genetic gain. So UC1 and EMBV are a good compromise for short-term genetic gain and UC2 to maintain diversity for a longer term's perspective.

#### Impact of different CSC on the mating design

We looked at the relation between the ranking of the parents (according to TBV in TRUE scenarios and GEBV in ESTIMATED scenarios), and their contribution to the mating plan, in terms of number of progenies and crosses, depending on CSC (Supplementary Figs. 2 and 3 Tables 2 and 3). Looking at parental allocation, mating designs based on PM systematically displayed the highest average genetic similarities between selected parents compared to other CSC (Supplementary Protocol 3 and Fig. 1). OHV and UC2 criteria displayed the lowest genetic similarities between recruited parents.

In CONSTRAINT scenarios, the contribution of the best parent to the progeny was around 4% for all CSC. At the opposite, in NO CONSTRAINT scenarios, the contribution of the best parent reached up to 25%.

OHV and PM are the 2 opposite extremes for all scenarios in terms of parental contribution, with OHV allocating less progenies to the top parents and UC2 intermediate. In CONSTRAINT, Selected, and TRUE scenarios, progenies were allocated to a more diverse set of parents compared to NO CONSTRAINT, Unselected, and ESTIMATED scenarios. The maximum is 25% of progenies attributed to the best parent for NO CONSTRAINT TRUE Selected scenarios using PM and PROBA, and the minimum is 7% for OHV. UC2 is in between, with 18% (14%) for Unselected (Selected) TRUE scenarios and 61% (55%) for ESTIMATED scenarios. A majority of progenies is attributed to the top 20 parents. For example, in CONSTRAINT scenarios, the top 20 parents produced 76% of progeny using criterion PM, 69–72% of progeny using PROBA, UC3, or EMBV, 51% using UC2, and 40% using OHV (Supplementary Table 2 and Fig. 2).

Looking at the distribution of contributions to crosses, it is not PM but UC3 and PROBA that allocate the highest number of progenies to top crosses. OHV select more crosses resulting in higher genetic diversity. UC2 is intermediate.

#### Impact of diversity constraints on selected progeny

The constraints that we used (C2: a maximum of 60 progenies per cross; C4: a maximum of 250 progenies per parent; C5: a minimum number of 100 parents recruited; C6: highly related lines could not be crossed) increased the genic variance by 10% (8–15% depending on the CSC and scenario) in the new breeding population and reduced the mean genetic value by 5% (4–8%; Table 1). Incidentally, the constraints also reduced the genetic value of the top progenies by around 2% for all CSC and up to 8% when using PM.

**Table 1.** Impacts of constraints in terms of genic variance and genetic gain for the top 7% progeny of the whole mating design and for the best progeny value.

Marker effects	TRUE		ESTIMATED	
Starting population	Unselected	Selected	Unselected	Selected
Genic variance of the best 7% progenies	**PM + 12%** Other CSC + 9%	**PM + 11%** Other CSC + 8%	**PM + 15%** Other CSC + 12%	PM + 13% Other CSC + 13%
Genetic gain of the best 7% progenies	PM: −8% **Other CSC −6%**	PM: −8% **Other CSC −5%**	**PM: −4%** Other CSC −5%	PM: −4% Other CSC −4%
Value of the best progeny	PM −8% **Other CSC −2%**	PM −8% **Other CSC −2%**	PM −4% **Other CSC −2%**	**PM −1%** Other CSC −2%

The values were computed as $\;\frac{\mathrm{value}\;\mathrm{in}\;\mathrm{CONSTRAINT}-\;\mathrm{value}\;\mathrm{in}\;\mathrm{NO}\;\mathrm{CONSTRAINT}}{\mathrm{value}\;\mathrm{in}\;\mathrm{NO}\;\mathrm{CONSTRAINT}\;}$ for each metric and each CSC and then averaged over the 30 genetic architectures. Values in bold represent CSC showing the most desirable response in the CONSTRAINT scenarios.

The CONSTRAINT scenarios had a significant negative effect on the TBV of the 7% progenies and the best progeny and positive effect on genic variance in top progenies, especially when using PM. This could be explained by the fact that the selected crosses using CONSTRAINT are suboptimal compared to NO CONSTRAINT in terms of genetic gain by forcing a minimum level of diversity in parents. For NO CONSTRAINT scenarios, the algorithm assigns a maximum number of progenies (60) to the 55 best crosses, while for CONSTRAINT scenarios, the objective function maximizes the sum of CSC of all selected crosses, with a limit of 250 progenies per parent for the whole design. Although selected parents were stable between different runs of a same scenario, the mate allocation seemed random with CONSTRAINT using PM or EMBV compared to other CSC (see the low percentage of crosses that were similar in 2 independent mating design optimizations from the same sets of parents in Supplementary Table 1).

**Fig. 4.**
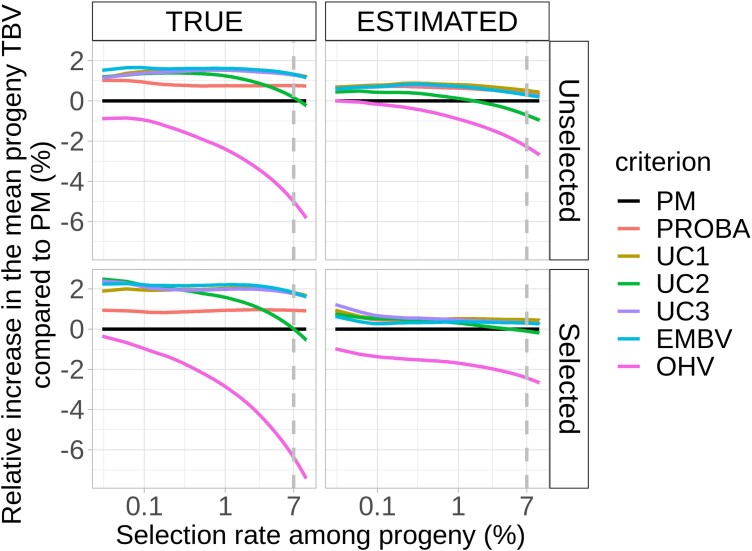
Relative increase in the mean TBV of selected progeny compared to progeny using the PM criterion for NO CONSTRAINT scenarios.

We observed an increase in the additional gain provided by CSC compared to PM in CONSTRAINT as compared to NO CONSTRAINT scenarios. For example, in the TRUE + CONSTRAINT scenarios, the additional gain using CSC was 2-fold higher in comparison to the TRUE + NO CONSTRAINT scenarios (Fig. 4).

Despite the fact that the difference between PM and other CSC was reduced for NO CONSTRAINT scenarios, alternative CSC still appeared to be much more advantageous compared to PM in providing high-value progenies. For the TRUE + Unselected scenario, EMBV provided the best progeny (relative increase compared to PM = 1.5% ± 1.6); for the TRUE + Selected scenarios, UC2 provided the best progeny (2.9% ± 2.4); for the ESTIMATED + Unselected scenarios, PROBA was the best criterion (0.7% ± 2.3); and for the ESTIMATED + Selected scenarios (closer to breeding programs), UC3 was the best criterion (1.3% ± 1.9).

## Discussion

### Rank of CSC for genetic gain and diversity management

Several CSC have been proposed to rank the crosses that are focused on different properties of the right-hand tail of the predicted distribution of progeny breeding values: UC (expected mean value of top progeny), PROBA (expected percentage of progeny with genetic value higher than a threshold), EMBV (best progeny value among *N* progenies), and OHV (best theoretical progeny value). One goal of the present study was to rank CSC based on their ability to provide superior short-term genetic gain but also to assess their impact on genetic diversity management. With perfect marker effect estimation (TRUE scenarios), mating designs optimized using progeny variance estimates using UC1, UC2, or EMBV provided superior genetic gain and diversity in top progeny than mating designs solely optimized with regard to parental breeding values. With estimated marker effects (ESTIMATED scenarios), UC1, UC2, and EMBV maintain a level of genetic diversity significantly superior while maintaining genetic gain.

The PM criterion served as a reference. For all scenarios, alternative CSC (except OHV) provided superior genetic gain in top progeny when selection was stringent. The OHV criterion was associated with a minor genic variance loss but also the lowest genetic gain. The potential of OHV to maintain genetic diversity had already been demonstrated by Daetwyler *et al*. (2015). PROBA, UC3, UC1, and EMBV criteria showed the highest genetic gain, whereas there was a genic variance loss close to that observed using PM. UC2 presented intermediate genetic gain and genetic diversity.

This study tested the interest of 2 CSC, PROBA and UC3, adapted from recent literature. The PROBA criterion, as described by Wellmann (2019), ranked crosses based on their probability of producing progeny superior to the best parental line. PROBA provided among the highest elite progenies for all scenarios (Fig. 2), except for TRUE + Unselected and NO CONSTRAINT scenarios (Fig. 4) where UC (criterion based on the expected superior quantile value of the progeny distribution) worked better. Note that a threshold must be set for the PROBA criterion. In this study, we opted to set this threshold according to the genetic value (TBV for TRUE scenarios or GEBV for ESTIMATED scenarios) of the best parental line. Different thresholds could be tested. Wellmann (2019) proposed to set the threshold based on historical genetic gain. Note that if the threshold is too high (or too low) compared to the expected progeny distributions (for a cross population), most crosses will have a 0 (or 1) PROBA value, which necessitates tuning this criterion for each trait and material.

The UC3 criterion aims to maximize the expected value of the 7% best progenies of the whole mating design. It is a direct application of the index 5 criterion concept tested in Bijma *et al*. (2020) but with no numerical approximation, thereby increasing the computation time. In terms of genetic gain, the UC3 criterion was among the best CSC for NO CONSTRAINT scenarios and TRUE + Unselected + CONSTRAINT scenarios. Note that for UC3 and EMBV, we could not use linear programming as for other CSC, so it was much more compute intensive. For instance, it took less than 10 min to optimize a mating design using 35k candidate crosses (with preselection of the 10% crosses with the highest PM), around 5 h to choose between 350k crosses (no preselection) using linear programming and around a day for UC3 or EMBV to reach reasonable convergence with our homemade GA.

In conclusion, according to the pareto front in Fig. 3, all criteria are superior to PM in terms of genetic gain, genetic diversity, or both, except UC3 in ESTIMATED + Selected scenarios. UC1 and UC2 criteria are a good trade-off for quick genetic gain optimization while maintaining genetic diversity. UC1 provides always superior or equal genetic gain compared to PM as well as superior diversity, while other criteria are better either for gain or for diversity. UC2 is most often superior to PM for both gain and diversity in TRUE scenarios, superior in terms of diversity, and equivalent in terms of genetic gain in ESTIMATED scenarios. So UC1 is a good choice for breeding programs that seek to maximize gain with no extra loss of diversity, and UC2 is a good choice for breeding programs focusing on maintaining diversity (prebreeding) without impacting genetic gain.

### Factors influencing the added value of CSC compared to PM

The major issue in genomic predictions is the precision of the estimation of marker effects, which depends on the size of the TP and its relevance of the TP (relatedness between the training and the prediction sets), the accuracy of phenotypes (number of locations, correction for spatial heterogeneity, and GxE…), and the genomic prediction models. The TRUE scenario provides information about the maximum potential of CSC if the TP is optimal and marker effects are perfectly estimated. Only if the relative performances of CSC in this ideal scenario are convincing, it is worth testing it in more realistic simulated breeding programs. This is a reference value that tells us how optimal is our design, on which parameters we can work to improve our predictions and the maximum gain we can ever get.

According to Figs. 2 and 4, the relative increase in progeny TBV for CSC based on progeny variance estimation was significant for TRUE scenarios but not for ESTIMATED scenarios. For TRUE scenarios, CSC were more efficient for Selected compared to Unselected scenarios. Two nonexclusive factors could explain these results: progeny variance estimation accuracy and progeny variance variability of candidate crosses.

#### Progeny variance estimation accuracy

First, CSC based on progeny SD estimates $(\hat{\sigma })\;$ was hampered by higher estimation error than the conventional PM criterion based solely on progeny mean estimates $\hat{PM}$ (Table 2). The correlation between estimated ($\hat{\sigma }$) and true SD (*σ*) was on average 4–22 points lower than the correlation between the estimated ($\hat{PM}$) and true PM. Note that both $\hat{PM}$ and $\hat{\sigma }$ accuracies were higher in ESTIMATED + Unselected populations than in ESTIMATED + Selected populations. It is hard to determine if it is due to the lower heritability in Selected populations (because the environmental variance was set as constant during in silico breeding) or the negative correlation between QTLs (Bulmer effect). Heritability was 0.4 in Unselected scenarios and 0.3 in Selected scenarios. Our hypothesis is that it cannot explain alone a diminution of correlation between estimated and true SD of a factor 2 (0.41 for Unselected and 0.16 for Selected scenarios). To prove that the negative relationship between QTL due to Bulmer effect in Selected scenarios explains this difference, we will have to fix heritability between Selected and Unselected scenarios in our next simulation papers.

**Table 2.** Correlation of the expected mean progeny estimate $\hat{\mathrm{PM}}$ and progeny SD $\hat{\sigma }$ compared to their true PM and σ values.

Population	$\hat{h^{2}}$	cor$\left(\hat{\mathrm{PM}},\mathrm{PM}\right)$	cor$\left(\hat{\sigma },\sigma \right)$
Unselected	0.4 ± 0.06	0.45 ± 0.05	0.41 ± 0.07
Selected	0.3 ± 0.03	0.38 ± 0.04	0.16 ± 0.07

Marker effects were estimated using GBLUP for Selected and Unselected scenarios. Values were computed on the 10% crosses with the highest PM. Heritability was computed as the ratio between twice the genetic variance parameter estimated by GBLUP and the phenotypic variance.

The lower accuracy of progeny variance estimates compared to genetic values has been reported in many studies (Lian et al. 2015; Neyhart and Smith 2019; Adeyemo and Bernardo 2019; Santos *et al*. 2019; Wolfe *et al.* 2021). Factors influencing the GEBV estimation accuracy, e.g. phenotyping quality, experimental design, statistical model used to take environmental effects into account, and the genetic relationship between the candidate and TP, probably impact progeny variance estimation accuracy as well. Concerning GEBV estimation, the different GS models tested in the literature usually lead to slight or moderate improvement in GEBV accuracy for quantitative traits while sometimes providing a significant improvement when trait variations were controlled by a few heterogenous QTLs (Daetwyler *et al*. 2008; Heslot *et al*. 2012). However, for progeny variance estimation, Bayesian models may markedly improve the accuracy for quantitative traits compared to the GBLUP model because of their ability to take into account the error in marker effect estimates. For example, Lehermeier *et al*. (2017) suggested using a Markov chain Monte Carlo (MCMC) algorithm to calculate the posterior mean of progeny variance (Lehermeier *et al*. 2017; Sorensen *et al.* 2001). In matrix notations, the progeny variance is calculated as ${\hat{\beta }}^{\prime }V_{ij}\hat{\beta }$, where $\hat{\beta }$ is the vector of estimated marker effects and $V_{ij}$ is the variance–covariance matrix of marker genotypes of the progeny derived from the cross between $\mathrm{Paren}t_{i}$ and $\mathrm{Paren}t_{j}$. The MCMC algorithm allows estimation of the posterior distribution of ${\hat{\sigma }}^{2}$ by averaging the product ${\hat{\beta }}^{\prime }V_{ij}\hat{\beta }$ for each sample of the posterior distribution of $\hat{\beta }$ [posterior mean variance (PMV) estimates]. Such PMV estimates were shown to be more accurate in estimating the true progeny variance. For instance, in simulations run by Lehermeier *et al*. (2017), for *h*^2^ = 0.4 with a 100–600 TP size range, the bias in the PVM estimate of progeny variance $\left((\hat{\sigma^{2}}-\sigma^{2})/\sigma^{2}\right)$ ranged from 0.06 to 0.21, while the correlation with the true value ranged from 0.58 to 0.65. This was much more accurate than what we obtained with our data for a similar scenario (Unselected + ESTIMATED, *h*^2^ = 0.4, training set size = 835, GBLUP model) with an average −0.82 ± 0.04 bias and 0.41 ± 0.07 correlation. Another strategy for estimating marker effects is to use selection models such as Bayesian Lasso that basically remove markers having very minor effects. In Santos *et al*. (2019) and Tiede *et al*. (2015), the Bayesian Lasso model provided more accurate marker effects and progeny variance estimates than GBLUP, but this was not the case in Yao *et al*. (2018). Finally, other GS models could be interesting to test with regard to increasing the progeny variance estimate accuracy, e.g. models using haplotypic blocks instead of markers (Cole and VanRaden 2011; Bonk *et al*. 2016). The idea is that combinations of alleles in haplotypic blocks may be better estimated (if present in the TP) than individual SNPs and segregate as a block in progeny. For bread wheat, the recombination hotspots described in Danguy des Déserts *et al*. (2021) could be used as haplotype block separators, for instance.

#### Progeny variance variability of candidate crosses

The benefits of CSC based on progeny variance estimation also highly depend on the ratio between the progeny SD and progeny mean variance *t* = var(*σ*)/var(PM). To understand why, let us follow the reasoning of Zhong and Jannink (2007) based on an example with the UC criterion: the expected value of the superior fraction *q* of the progeny of a cross is computed as UC = PM + i*σ, with *i* being the selection intensity corresponding to the selected quantile *q*. The variance of UC values is thus equal to var(PM) + *i*^2^*var(*σ*) + 2*i*cov(PM, *σ*). We can thus hypothesize that the lower the *t* ratio, the more the UC variance could be explained by the PM variance. In other words, when the *t* ratio is low, the genetic values of parents (e.g. ∼PM) drive the expected superior progeny value. Hence, all CSC tend to select the same crosses, leading to a low additional genetic gain of alternative CSC over PM. For ESTIMATED scenarios (*t* = 2–3%), the *t* ratio was 4-fold lower than for TRUE scenarios (*t* = 6–11%) (Table 3). As expected, the mating designs in our analysis were more similar when the *t* ratio decreased. Supplementary Fig. 4 shows a higher pairwise correlation of CSC for ESTIMATED compared to TRUE scenarios. Moreover, the proportion of shared parents between mating designs obtained with different CSC increased for ESTIMATED scenarios, as well as the genetic similarity between recruited parents (Supplementary Protocol 3 and Figs. 1 and 5). As the *t* ratio is highly decisive for the added value of alternative CSC over PM, it is important to properly estimate the progeny variance, e.g. using previously described models (PMV and Bayesian Lasso).

**Table 3.** Ratio between the variance of progeny SD *σ* and the progeny expected mean PM for various scenarios.

TRUE var(*σ*)/var(PM)		ESTIMATED var($\hat{\sigma }$)/var($\hat{\mathrm{PM}}$)	
Unselected	Selected	Unselected	Selected
6% ± 1%	11% ± 2%	3% ± 0.1%	2% ± 0.2%

This was calculated for the 10% crosses with the highest PM.

Bijma *et al*. (2020) tested the added value of several CSC for populations with different *t* ratios, and populations with a high *t* ratio systematically showed higher alternative CSC benefits. We can list some population types that are expected to have a high *t* ratio and would thus be worthy of CSC implementation. First, selection can increase *t* ratio. In Bijma *et al*. (2020), it was hypothesized that in the context of an infinitesimal model and infinite populations, the progeny variance does not change over generations. However, in our simulations, e.g. in finite populations with a finite number of causal loci, var(PM) was reduced by selection (3-fold lower for Selected scenarios compared to Unselected scenarios), as well as var(*σ*) but at a lower extent (1.4-fold lower for Selected compared to Unselected scenarios). The TRUE *t* ratio thus increased, along with the expected benefits of CSC based on progeny variance estimation. Second, structured populations can also lead to high *t* ratios. Structured populations arise when crossing elites with genetic resources (GR) from different genetic groups, in prebreeding programs for instance, or to a lesser extent when crossing elite parents to elites from different breeding companies. When crossing parents from 2 highly differentiated populations, the *t* ratio may increase because of a higher magnitude of var(*σ*). Genetic differentiation leads to higher polymorphism between parents from different genetic groups (Wahlund effect) and among progenies and thus higher progeny variance. According to Bijma *et al*. (2020), structuration in plants may explain the negative correlation between PM and *σ* reported in several publications in maize (Bernardo 2014; Mohammadi *et al*. 2015), bread wheat (Lado *et al*. 2017), and barley (Abed and Belzile 2019; Neyhart and Smith 2019). In our case, we also observed a negative relationship between PM and *σ* in INRAE-AO data analyses and simulations. The negative relationship was higher in Unselected scenarios (Supplementary Fig. 4). A negative correlation indicates that crosses with a low to medium PM (elite*GR) had a higher progeny variance than crosses between elite parents. In these situations, ranking crosses according to CSC based on progeny variance estimation may thus be very useful for increasing genetic gain.

We hypothesized that the genetic structure of the population and accuracy of progeny variance estimates were the 2 factors explaining high *t* ratios and in turn high benefits of alternative CSC (Lehermeier *et al*. 2017; Yao *et al*. 2018). Lehermeier *et al*. (2017) used a maize Nested Association Mapping population built with European dent landraces (Bauer *et al*. 2013) crossed to 1 elite accession, leading to a family-structured progeny. The ratio $\mathrm{var}(\hat{\sigma })/\mathrm{var}(\hat{\mathrm{PM}})$ was on average 14% (*h*^2^ = 0.2 and *h*^2^ = 0.6). The ratio obtained in our study using elite bread wheat material ranged from 2 to 11% (Table 3). In Lehermeier *et al*. (2017), the genetic gain provided by UC compared to PM was superior to 0.2 genetic SD $\left(\sigma_{g}\right)$ at a selection rate inferior to 10%. This was 5-fold higher than our best results for the 7% top progenies under similar scenarios (*h*^2^ = 0.4; Unselected + TRUE scenarios: genetic gain = 0.04 $\sigma_{g}$; Unselected + ESTIMATED: genetic gain = 0.035 $\;\sigma_{g}$). Yao *et al*. (2018) used bread wheat crosses involving Chinese and Australian lines that were likely very differentiated and thus likely associated with a high *t* ratio. In Yao *et al*. (2018), the genetic gain provided by UC was 0.06 $\;\sigma_{g}$ at *h*^2^ = 0.3, 0.08 $\sigma_{g}\;$ at *h*^2^ = 0.5 $\sigma_{g}\;$, and 0.13 $\;\sigma_{g}$ at *h*^2^ = 0.8, for a selection rate ranging from 1 to 10%. This level was similar to what we observed for TRUE + Unselected scenarios and 2-fold higher than ESTIMATED + Unselected scenarios (0.035 $\sigma_{g}$).

Note that we did not observe any specific structuration in our founders (*n* = 835 winter wheat inbreds from INRAE and AO; Supplementary Fig. 6).

### Trade-off between genetic gain and genetic diversity

The breeder's equation implies that genetic gain is proportional to the selection intensity and genetic variance. However, the theory also predicts that in an isolated breeding program, without extrinsic germplasm introduction, each selection step is associated with a reduction in genetic variance. Genetic gain in successive generations would thus be expected to decrease and finally converge to 0 when there is no longer genetic variance in the breeding population (Jannink *et al*. 2010). This phenomenon is faster with GS, which decreases the generation interval, increases the selection intensity if the accuracy is high, and increases the probability of selecting related individuals (Clark *et al.* 2011; Pszczola *et al.* 2012).

Several methods have been suggested in the literature to optimize genetic gain while managing genetic diversity. For example, several authors (Jannink *et al*. 2010; Goddard 2009; Hayes *et al*. 2009) suggested giving more weight to rare and favorable alleles when computing GEBV on candidate parents [weighted GS (WGS)]. Goiffon *et al.* (2017) suggested selecting a set of candidate parents that bear at least 1 copy of all beneficial alleles. Alternatively, the optimal contribution selection (Meuwissen 1997) or optimal cross selection (OCS; Kinghorn *et al*. 2009; Allier, Moreau, *et al*. 2019) methods optimize parental contributions in order to maximize genetic gain while constraining average pairwise inbreeding (Falconer and Mackay 1996, reviewed in Woolliams *et al*. 2015). In plants, these methods have been adapted to inbreds by Allier, Moreau, *et al*. (2019) to maximize genetic gain while limiting the loss of mean expected heterozygosity in future progeny: $\mathrm{He}=1\text{–}\frac{1}{2}c^{\prime }\Phi c$, where *c* is the contribution of parents to progeny and Φ is the identity by state matrix (Allier, Moreau, *et al*. 2019). The expected genetic diversity He is determined by the distribution of progenies among candidate parents (*c* values) and the genetic similarity of parent Φ. As a rule of thumb, the overuse of the few (best) parents (Wray and Thompson 1990) and the use of highly similar parents have a negative impact on the expected heterozygosity. In this study, instead of controlling He in progeny, we imposed commonly used constraints on the mating design with a minimum number of parents and crosses and avoiding crossing similar parents. The next step is to add to our pipeline OCS in order to explicitly take into account coancestry of parents. In INRAE-AO material, we showed that setting empirical constraints on parental contributions actually had little impact on genetic gain but highly preserved the genetic diversity.

### Practical breeding implications

Maximizing genetic gain and genetic diversity by doing grid search on parameters such as the cross selection criteria to use, the total number of crosses, the number of progenies per cross, and the number of parents, considering a fixed budget, is an interesting but extensive work that has to be optimized by each breeder taking into account its economic context and germplasm.

Our priority here was to compare published and adapted CSC for short-term genetic gain and genetic diversity management and make the code available for breeders. CSC based on progeny variance estimation are more interesting in TRUE + Selected scenarios with CONSTRAINT, which corresponds to a real breeding program scenario. Their interest necessitates to get as close as possible to the TRUE scenario by optimizing marker effect estimates and TP. According to Fig. 3, UC1 is a good compromise for short-term genetic gain with limited loss of genetic diversity, and UC2 should be preferred for maintenance of genetic diversity in a prebreeding context for instance (Table 4).

**Table 4.** Relative advantage of CSC compared to PM.

CSC	Genetic gain	Genetic diversity
PROBA	+++	
UC1	++	+
UC2	+	++
UC3	+++	
EMBV	++	+
OHV	−	+++

Further simulations with more generations would be necessary to quantify the long-term genetic gain and diversity using CSC for different t ratios.

Per se and cross genetic value predictions will not replace breeder's expertise. But when several parameters including genomic predictions support the quality of an individual or a cross, 1 strategy can be to bet and invest on those lines or crosses. For best lines, the breeder can decide to accelerate the selection process using double haploids and diminish the number of years of evaluation before registration. For mating plans, the breeder can decide to produce a larger number of progenies for crosses with high UC or PROBA, in order to be sure to get an outstanding progeny (in the queue of the predicted distribution). In practice, the breeders may not allow progeny size to vary widely among crosses as this is the case in our simulations. But they can decide to have regular size progenies for most crosses and extended sizes for a few crosses with high UC1, UC2, or PROBA. It would be interesting to add to the pipeline the estimation of the size of the progeny to assure the achievement of a realistic value for PROBA, especially when the possibility to include several traits will be included.

Some more work is also necessary to optimize the threshold we choose for PROBA. It must be in the range of variation of the putative parents in order to be realistic. Putting an extreme threshold on PROBA would be equivalent on focusing on the extreme tails of the progeny distribution. When the threshold is too high (no cross can provide progeny that satisfies the constraints), all crosses have a PROBA close to 0. When the threshold is too low, all crosses provide a PROBA close to 1. So, the risk choosing extreme thresholds is that PROBA will not be discriminant or the probability to actually sample such extreme progeny in selected crosses would be too low with our constraints on progeny size. In our case, setting the threshold as the value of the best line in the TP gave a discriminant PROBA.

Regarding computation time, we provide a solution to quickly estimate progeny variance in Supplementary Protocol 1. On a usual laptop, this solution allows to estimate progeny variance of a diallel of 835 parents in 2 h. Regarding time for optimization of mating plans, linear programming (free software lp_solve on Linux) needed only 10 min to optimize mating plans of 35k candidate crosses. So once a pipeline is established, computation time is not a problem.

### Conclusion

For an elite winter bread wheat breeding program, crossing parents with the highest genetic values is likely not the best means to maximize the usefulness of progeny. Alternative CSC that take progeny variance estimation into account could provide better elite progeny and improve the mean population value while maintaining more genetic diversity. UC1 is a good compromise for short-term genetic gain with limited loss of genetic diversity, and UC2 should be prefered for maintenance of genetic diversity that could be of interest for longer-term genetic gain, in a prebreeding context for instance. However, the efficiency of these alternative CSC depends on the progeny variance estimation accuracy, which requires some improvement. The size and quality of the TP should be increased as well as marker effect estimates. Moreover, the interest of CSC compared to mean parental value increases when the *t* ratio var(*σ*)/var(PM) among crosses increases and should be evaluated in a prebreeding program.

## Supplementary Material

jkad195_Supplementary_Data

## Data Availability

Genotypes (GenotypingData.txt) and phenotypes are available in the INRAEDataverse repository (https://data.inra.fr/) with the following links: https://doi.org/10.15454/AABGO7 and https://doi.org/10.57745/BSHZKV. Scripts to reproduce all the results are available on Github (https://github.com/aldanguy/mating_plans_bread_wheat). Supplemental material available at G3 online.
