# Supplementary material for: Comparison of genomic-enabled cross selection criteria for the improvement of inbred line breeding populations
Source: G3 (Bethesda). 2023 Aug 25;13(11):jkad195. doi: 10.1093/g3journal/jkad195 (PMC10627264; doi:10.1093/g3journal/jkad195)
Supplement: jkad195_Supplementary_Data [file jkad195_supplementary_data.docx]

# **I) Supplementary Protocols**

## I.1) Supplementary Protocol S1: Fast computation of progeny variance

Let 𝑁 be the number of candidate parents (not necessarily inbred) and M be the number of markers. There are N(N-1)/2 candidate crosses. Progeny variance derived from the F1 of the cross between parent i and j can be formulated as

$\sigma_{ij}^{2}$ = β’$D^{ij}\beta$ **[1]**

Where β is the vector of marker effects (size M*1) and $D^{ij}$ is the variance-covariance matrix of progeny genotype specific to the i*j cross (size M*M).

Alternatively, progeny variance can be formulated as:

$\sigma_{ij}^{2}$ = ($\beta^{i}$− $\beta^{j}$)’ $D^{(1)}$ ($\beta^{i}$− $\beta^{j}$) **[2]**

Where $\beta^{i}$ is the vector of marker effects for parent i (size M*1). Genotypes are encoded 0, 1 and 2, i.e. the number of alleles displaying the alternative effect $\beta^{i}$at each locus k. If the parent i is homozygous for the alternative allele (genotype 2), the k-^th^ element of vector $\beta^{i}$ is equal to 2*$\beta_{k}$, if parent i is heterozygous (genotype 1), the k-^th^ element is equal to $\beta_{k}$, and when parent i is homozygous for the ‘wild’ allele (genotype 0), the k-^th^ element is equal to 0.

The variance-covariance matrix of gamete genotypes is denoted $D^{(1)}$ (size M*M), and is common to every i*j cross (does not depend on the genotypes of the cross). For inbred progeny, diagonal elements of $D^{(1)}$ are equal to 0.25. For doubled haploid (DH) progenies, off-diagonal elements ${D^{(1)}}_{kl}$= 0.25 (1−2$r_{kl}$), while for RILs F5 progenies ${D^{(1)}}_{kl}=0.25*($ $1-2{r_{kl}}^{5}-(0.5($1-2$r_{kl}{))}^{5}$)))

Let us develop formula **[2]**:

$\sigma_{ij}^{2}$ = ($\beta^{i}$− $\beta^{j}$)’ $D^{(1)}$ ($\beta^{i}$− $\beta^{j}$)

= $\beta^{i'}D^{(1)}$ $\beta^{i}$+$\beta^{j'}D^{(1)}$ $\beta^{j}$ −2 $\beta^{i'}D^{(1)}$ $\beta^{j}$ **[3]**

Let $\beta^{i}'$ = $\gamma^{i}'$ $\beta^{(2)}$, where $\gamma^{i}$ (size 1*M) is the vector of parent i genotypes (0, 1 or 2 depending on the loci). $\beta^{(2)}$ is a diagonal matrix (size M*M) whose diagonal elements $\beta_{kk}^{(2)}$ are equal to marker effect $\beta_{k}$.

Formula **[3]** becomes:

$\sigma_{ij}^{2}$ = $\gamma^{i}'$ $\beta^{(2)} D^{(1)}$ $\beta^{(2)}$ $\gamma^{i}$ + $\gamma^{j}'$ $\beta^{(2)} D^{(1)}$ $\beta^{(2)}$ $\gamma^{j}$ – 2 $\gamma^{i}'$ $\beta^{(2)} D^{(1)}$ $\beta^{(2)}$ $\gamma^{j}$ **[4]**

Let $D^{(2)}$ = $\beta^{(2)} D^{(1)}$ $\beta^{(2)}$ be the weighted variance-covariance matrix common to every cross i*j. The formula **[4]** becomes:

$\sigma_{ij}^{2}$ = $\gamma^{i}'$ $D^{(2)}$ $\gamma^{i}$ + $\gamma^{j}'$ $D^{(2)}$ $\gamma^{j}$ – 2 $\gamma^{i}'$ $D^{(2)}$ $\gamma^{j}$ **[5]**

Let us define $\mu^{i}$= $\gamma^{i}'$ $D^{(2)}$ $\gamma^{i}$ (size 1*1), and also define the vector $\tau_{i}'= - 2 \gamma^{i}' D^{(2)}$ (size 1*M) and $\kappa_{1}^{j}$ the index of elements of $\gamma^{j}$ equal to 1 and $\kappa_{2}^{j}$ the index of elements of $\gamma^{j}$ equal to 2. The quantity $\tau_{i}$′$\gamma^{j}$ (size 1*1) can simply be computed as $\tau_{i}$′$\gamma^{j}$ = $\sum_{\{l|lЄ\kappa_{1}^{j}\}} \tau_{il}$+2*$\sum_{\{l|lЄ\kappa_{2}^{j}\}} \tau_{il}$.

Example:

Take $\gamma^{j}$’= (2 2 0 1 0 0 2), so $\kappa_{1}^{j}$= (4) and $\kappa_{2}^{j}$ = (1, 2, 7), so $\tau_{i}$′$\gamma^{j}$ = $2*{\tau_{i}'}_{1}$+ 2* ${\tau_{i}'}_{2}$+ ${\tau_{i}'}_{4}$+ ${{2*\tau}_{i}'}_{7}$

Finally, formula **[5]** can be converted to

$\sigma_{ij}^{2}$ =$\mu^{i}$+$\mu^{j}$ + $\tau_{i}$′$\gamma^{j}$ **[6]**

Using formula **[1]**, we first have to compute one variance-covariance matrix $D^{ij}$ (size M*M) for each of the N(N-1)/2 candidate crosses, which is already a huge task when the number of candidate parents N and/or of markers M is high.

To avoid this, formula **[2]** could be used to compute a single variance-covariance matrix D (size M*M) common to every candidate cross. Let us say that D has been computed. Computing progeny variance $\sigma_{ij}^{2}$ with formula **[2]** would require 𝑁(𝑁−1) /2 by 𝑀 subtractions to compute ($\beta^{i}$ − $\beta^{j}$) for each candidate cross, and then 𝑀×(𝑀+1) additions or products to compute ($\beta^{i}$ − $\beta^{j}$)’D for one cross, and finally M+1 supplementary additions or products to obtain ($\beta^{i}$ − $\beta^{j}$)’D ($\beta^{i}$ − $\beta^{j}$) for one cross.

Let us define an addition (or subtraction) computation time $T_{a}$ and a product computation time $T_{p}.$ Computing progeny variance of each candidate cross with formula **[2]** requires a total computation time of:

(N(N−1) /2) ×M×(M+1) × $T_{a}$+ (N(N−1) /2) ×M×(M+1) × $T_{p}$ **[7]**

Formula **[3]** requires computation of the weighted variance-covariance matrix $D^{(2)}$ = $\beta^{(2)}$𝐷 $\beta^{(2)}$ common to every candidate cross. This matrix $D^{(2)}$ requires some additional computation compared to D, but it should be computed only once. Let us say $D^{(2)}$ has been computed. Formula **[3]** also requires computation of

- N terms $\mu^{i}$= $\gamma^{i}'$ $D^{(2)}$ $\gamma^{i}$ (one per candidate parent). Each term μ requires (M+1) $T_{a}$+ M(M+1) $T_{p}$ for a total computation time of N[M+1) $T_{a}$+ M(M+1) $T_{p}$].
- N(N-1)/2 terms $\tau_{i}$′$\gamma^{j}$(one per candidate cross). Each $\tau_{i}$′$\gamma^{j}$term requires computation of $\tau_{i}'= - 2 \gamma^{i}' D^{(2)}$ associated with a computation time of M$T_{p}$+ M$T_{a}$ and then $\tau_{i}$′$\gamma^{j}$ associated with a computation time of around M$T_{a}$ if all loci of the second parent j are homozygous for alternative allele, or otherwise heterozygous. Thus, these N(N-1)/2 terms $\tau_{i}$′$\gamma^{j}$ are associated with a total of N(N-1)/2[$\mathrm{MT}_{p}$+ 2M$T_{a}$]. In summary, formula **[6]** requires a computation time of

N[M+1) $T_{a}$+ M(M+1) $T_{p}$ ] + N(N-1)/2[$T_{p}$+ 2M$T_{a}$] **[8]**

Directly computing progeny variance for every candidate cross with formula **[1]** or **[2]** yields a computation time of around N²M², while using the piece-wise formula **[6]** yields a computation time around NM² + N²M. For any M or N value, the second method **[6]** is always faster than the first methods **[1]** or **[2]** because $N^{2}$𝑀 + 𝑁$M^{2}$ ≪ $N^{2}M^{2}$.

To gain even more computation time, the progeny variance $\sigma_{ij}^{2}$ for each chromosome should be computed independently [$\sigma_{ij c}^{2}$, where c is the chromosome] and then the variance of all chromosomes [$\sigma_{ij}^{2}$=$\sum_{c} \sigma_{ij c}^{2}$] should be summed. Indeed, chromosome segregation during meiosis is random and thus the expected recombination frequency for loci located on different chromosomes is 0.5. The off-diagonal elements of variance-covariance of gamete genotypes are thus equal to 0 when the loci are not located on the same chromosome.

## I.2) Supplementary Protocol S2: Genetic algorithm

Mating designs based on EMBV and UC3 require a heuristic algorithm to be optimized. We used a Darwinism-inspired genetic algorithm (GA). GA starts from a fixed-sized population of individuals representing candidate solutions. In our case, a candidate solution is a vector of $D_{ij}$. A selection process is applied to produce the most promising solutions. Some pairs of candidates are crossed to create children, while others are muted. The new solutions replace their parents in the next generation.

The candidate solutions ($D_{ij}$vectors) are initially generated randomly. In the following generations, in order to respect the constraints, new vectors (i.e. individuals) are corrected after every modification (crossover or mutation), and before evaluation.

- First, the number of parents is checked (Constraint C5): if too many parents are represented, some $D_{ij}$are randomly set at zero, if too few parents are selected, new Dijs are moved from zero to non-zero values.

- Second, the number of progenies derived from one parent is checked (Constraint C4). If it is too big, some Dijs are reduced to respect this constraint without violating Constraint C5.

- Third, if the number of different progenies (number of non-zero $D_{ij}$) is too small, new ones are added, if it is too large it is reduced (Constraint C3). This is the case if correction one and/or two are needed. This is done without violating Constraint C5. Respecting constraint C4 can be difficult in some cases: consequently, if the number of attempts to meet Constraint C3-C5-C4 exceeds the size of the parent population, Constraint C4 is released and, instead of accepting 250 progenies derived from the same parent, we increase the boundary to 251, and so on until the constraint can be respected.

- Fourth, $D_{ij}$values are modified when necessary to respect Constraint C2 without violating constraints C3, C4 and C5. Moreover, C4 can be released after a number of failed attempts exceeding the parent population size.

- Finally, the sum of $D_{ij}$is checked to meet the number of progenies chosen (Constraint C1). At this step, it can be hard to randomly modify $D_{ij}$values and respect constraints C5, C4, C3 and C2, and exactly reach the fixed value. C4 can once again be released if necessary.

If C4 has been released, the evaluation function in the GA is penalized: if x is the max number of progenies produced by a parent and x>250, the criteria presented in the previous section are multiplied by 250/x. With such penalization, the best GA population element observed at the end of the algorithm always meets Constraint C4 in our simulations.

One crossover randomly creates two children by randomly picking and recombining the $D_{ij}$from two candidates. The children are corrected to respect constraints C1 to C5.

Mutation exchanges randomly 1 to 5 progenies between two candidates. The mutated candidate solution is corrected to respect the constraints. The process is repeated randomly 1 to 10 times and the best solution is kept.

The sharing process imposes a distance constraint between candidate solutions. We defined a simple distance which only sums non-zero $D_{ij}$in one candidate solution that are zeros in the other one, and reciprocally.

We used the following values for the application:

-Population size: 100

-Number of generations: 400,000. This number of generations was necessary for the algorithm to converge and was experimentaly set for randomly Selected populations.

-Crossover rate: 30%

-Mutation rate: 50%

## I.3) Supplementary protocol S3: Genetic similarity of mating designs

Within each of the s = 16 scenarios (Unselected/Selected * TRUE/ESTIMATED * CONSTRAINTS/NO CONSTRAINT), the genetic similarity S between mating designs from two CSC a and b is computed as:

$$S_{s}^{CSC a CSC b}=\frac{1}{P}\sum_{p(s)} \frac{c_{p(s)}^{CSC a}'*G_{p(s)}^{(2)}* c_{p(s)}^{CSC b}}{\sqrt{c_{p(s)}^{CSC a} '*G_{p(s)}^{(2)}*c_{p(s)}^{CSC a}} * \sqrt{c_{p(s)}^{CSC b} '*G_{p(s)}^{(2)}*c_{p(s)}^{CSC b}}}$$

Where p(s) is the index of the parental population tested in the scenario s (P=30), $G_{p(s)}^{(2)}$ is the genomic relationship matrix from LDAK software and $c_{p(s)}^{CSC}$ is the vector (size Nparents * 1) of the number of progenies allocated to each parent.

To measure the intrinsic genetic similarity generated by a mating design, we computed the increase in similarity between parents recruited using CSC compared to parents recruited using PM, as follows:

$$S_{s}^{CSC a}=\frac{1}{P}\sum_{p(s)} \frac{c_{p(s)}^{CSC a}*G_{p(s)}^{(2)}* c_{p(s)}^{CSC a}}{c_{p(s)}^{PM}*G_{p(s)}^{(2)}* c_{p(s)}^{PM}}$$

# **II) Supplementary Figures**

**
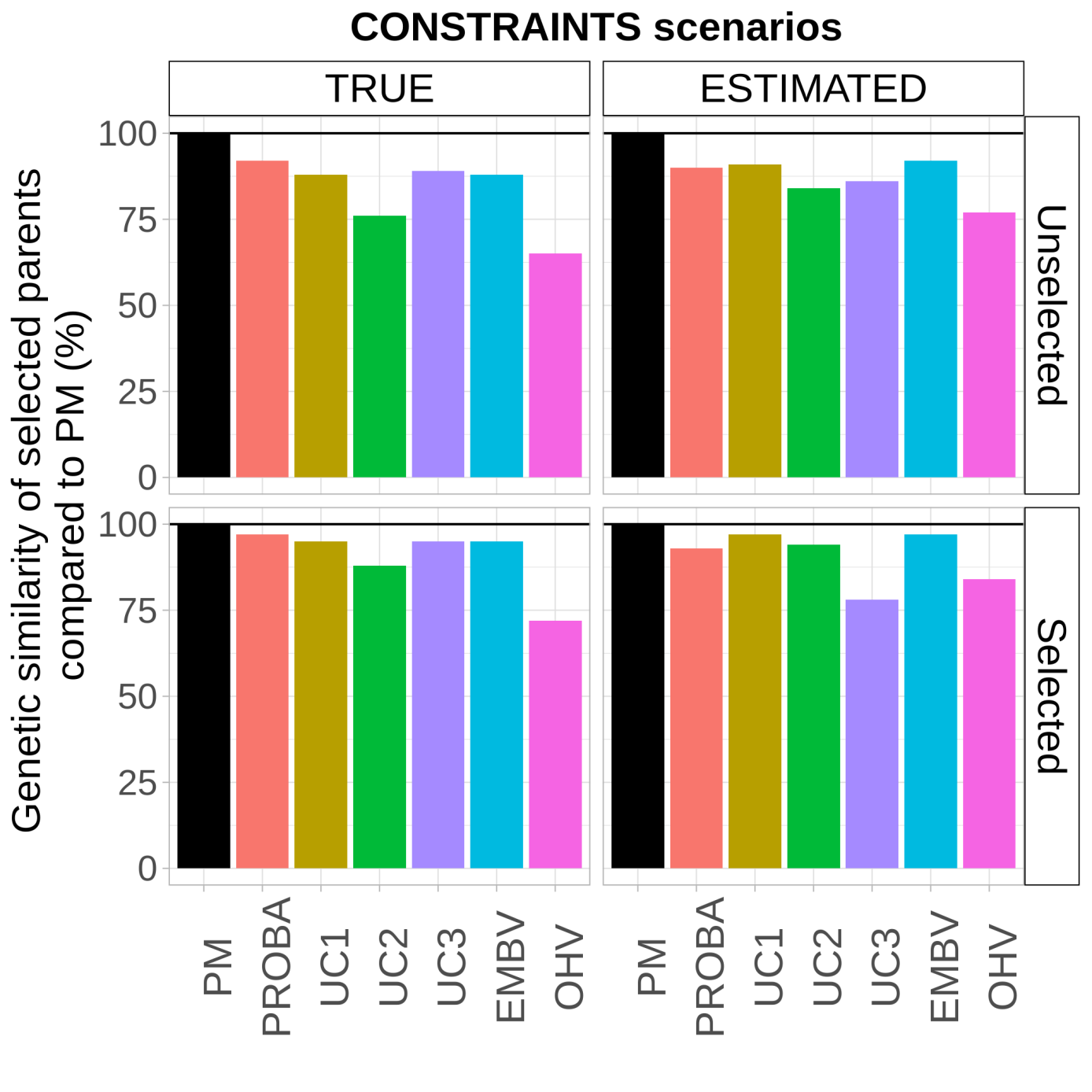
**

***Supplementary Figure S1: Genetic similarity of mating designs****, computed as the average parent variance-covariance weighted by their number of progenies using this CSC, divided by the average parent variance-covariance weighted by their number of progenies using the PM criterion, multiplied by 100 (****Supplementary Protocol S3****). Values below 100% indicate that the parents recruited when optimizing the mating design using this CSC are less similar on average than when using PM.*


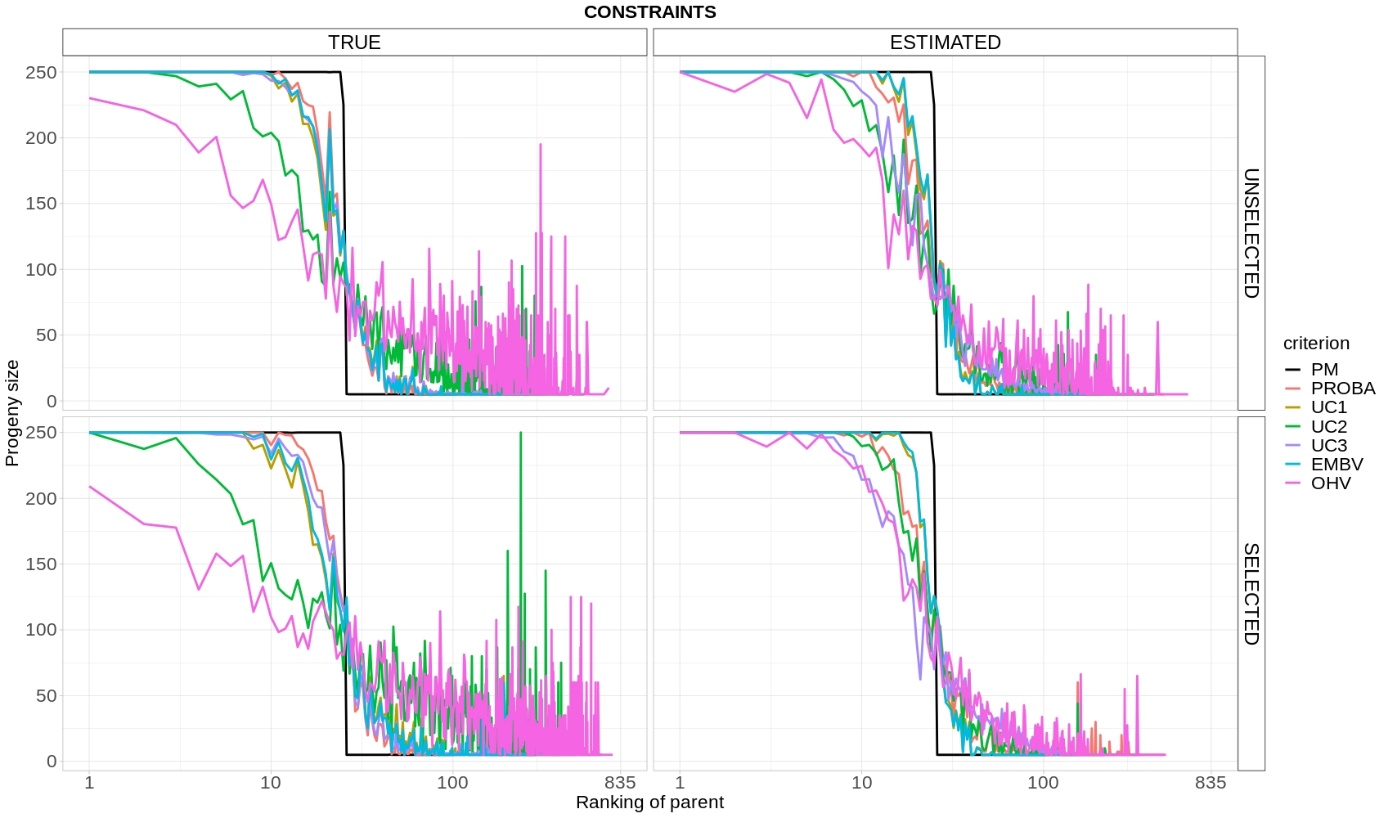


***Supplementary Figure S2: Progeny size and ranking of parents in CONSTRAINTS scenarios.*** *Ranking of parents is done using TBV in TRUE scenarios and GEBV in ESTIMATED scenario.*

*
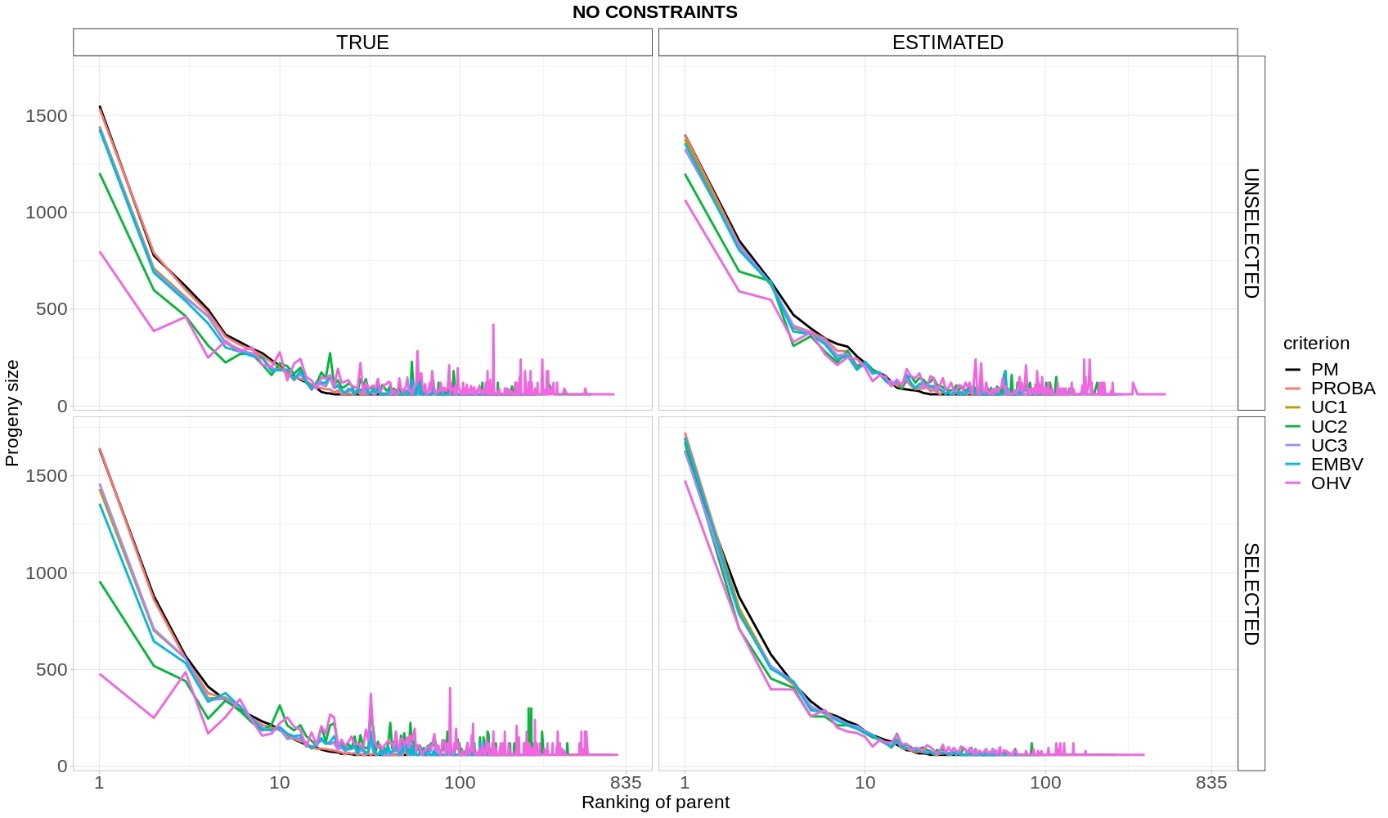
*

***Supplementary Figure S3: Progeny size and ranking of parents in NO CONSTRAINTS scenarios.*** *Ranking of parents is done using TBV in TRUE scenarios and GEBV in ESTIMATED scenario.*

*
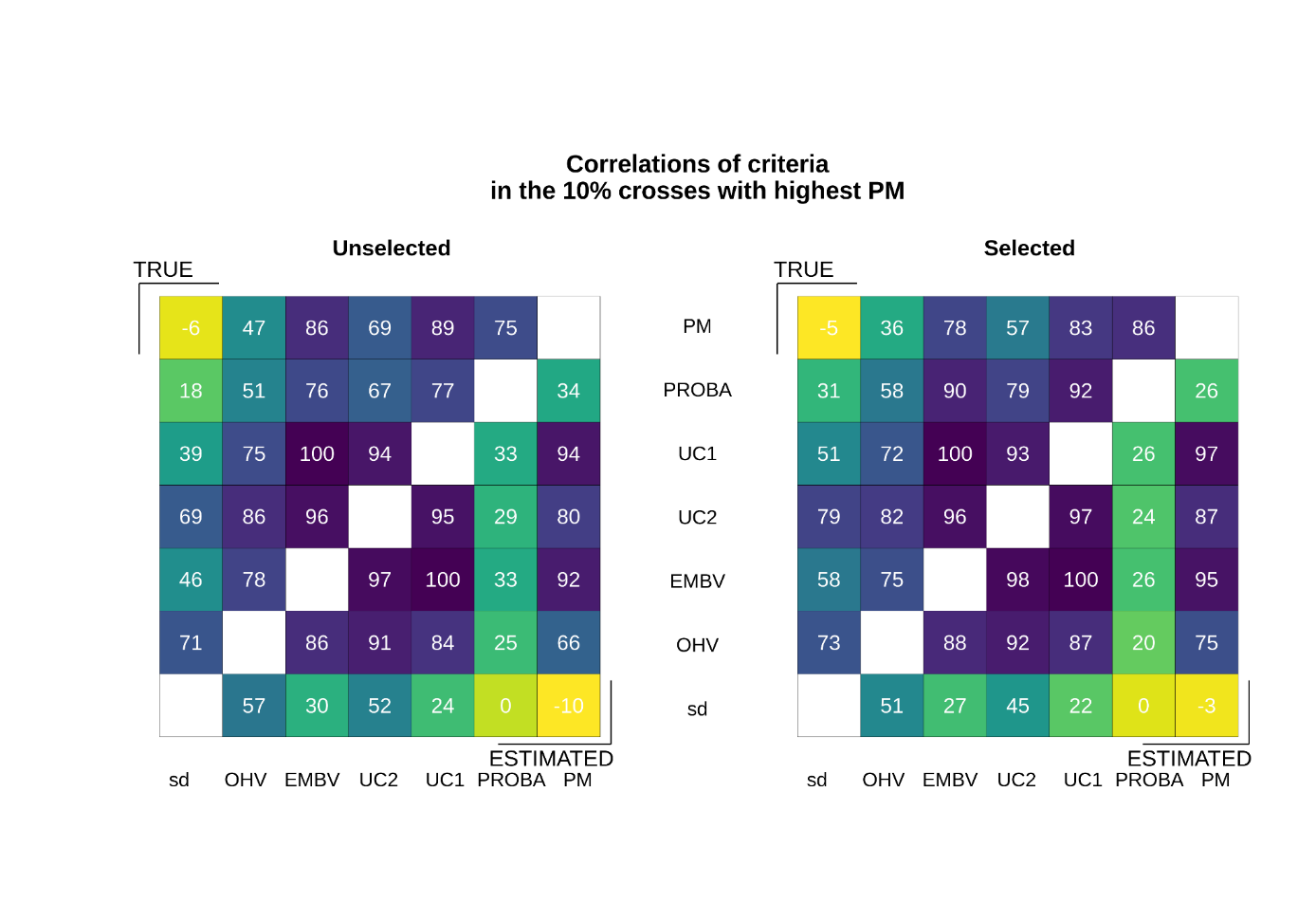
*

***Supplementary Figure S4: Pairwise correlations of CSC and progeny standard deviation (sd) in the 10% crosses with the highest PM.***

*
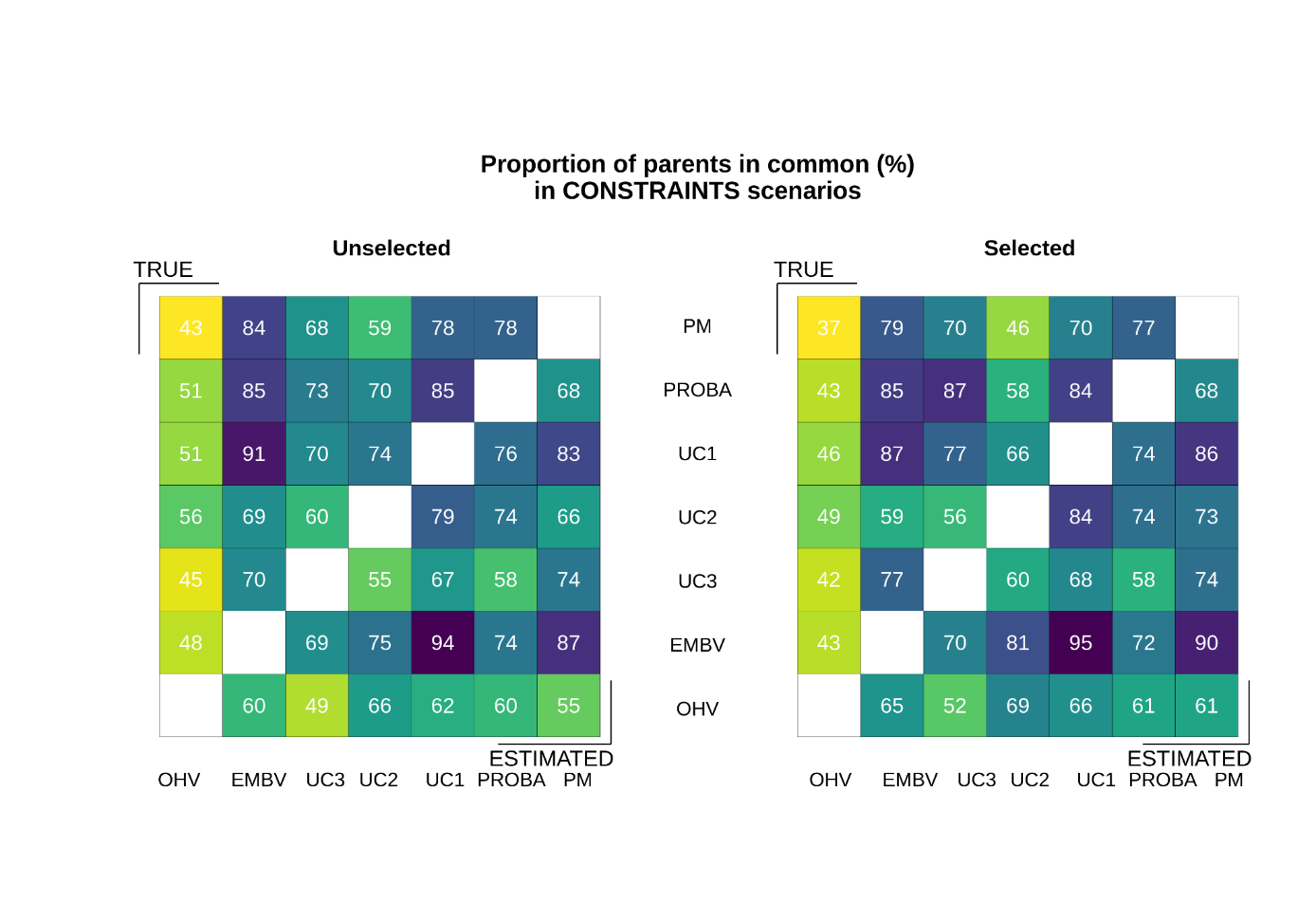
*

***Supplementary Figure S5: Proportion of shared parents****, computed as the number of separate parents selected in both mating designs divided by the total number of separate parents selected in at least one mating design.*

*
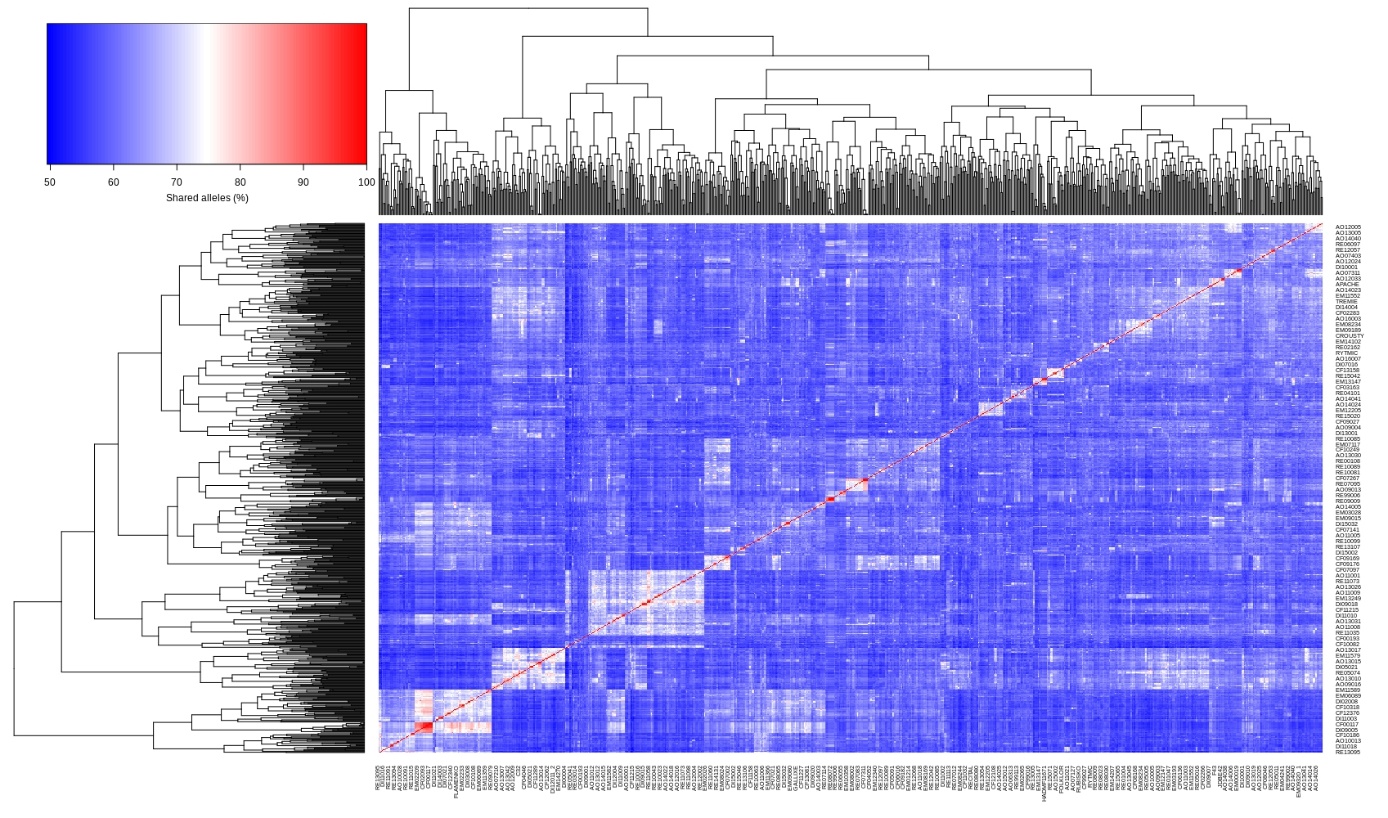
*

***Supplementary Figure S6: Proportion of shared alleles and clusterisation of the 835 bread wheat inbred genotypes from INRAE and AO.***

# **III) Supplementary Table**

|  | Parents in common | Crosses in common | Parents in common | Crosses in common | Parents in common | Crosses in common |
| --- | --- | --- | --- | --- | --- | --- |
| crosses | Pre-selected on PM | | No pre-selection | | Similarity of results with pre-selection and with no pre-selection | |
| **PM** | **100 ± 0** | **39 ± 3** | **100 ± 0** | **27 ± 1** | **100 ± 0** | **9 ± 4** |
| UC1 | 98 ± 0 | 97 ± 1 | 98 ± 2 | 97 ± 2 | 97 ± 1 | 95 ± 3 |
| EMBV | 89 ± 5 | 35 ± 5 | 74 ± 2 | 22 ± 1 | 67 ± 3 | 7 ± 1 |
| PROBA | 97 ± 3 | 97 ± 1 | 98 ± 2 | 96 ± 4 | 93 ± 2 | 90 ± 4 |
| UC2 | 99 ± 1 | 99 ± 1 | 100 ± 0 | 99 ± 2 | 95 ± 2 | 94 ± 3 |
| OHV | 100 ± 0 | 100 ± 0 | 100± 0 | 100 ± 0 | 80 ± 6 | 74 ± 8 |

***Supplementary Table S1: Proportion (%) of parents and crosses in common between mating designs of the same CONSTRAINT scenarios***

*Pre-selection of crosses was done by ranking all of the 350k candidate crosses based on PM and keeping the 10% (35k) best crosses. The mating design stability (e.g. shared parents/crosses) was studied in three populations from randomly chosen TRUE + Selected scenarios, and the average values as well as the standard deviations over the three populations are given. The first four columns give the similarity in the mating designs for two independent runs of the optimization algorithm (CPLEX for PM, UC1, UC2, PROBA and OHV, or a Genetic Algorithm for EMBV and UC3) from the same dataset of candidate crosses. The first two columns refer to a pre-selected dataset, while the next two columns refer the full dataset. The last two columns compare results obtained from the pre-selected dataset and the results obtained from the full dataset. The proportion of parents (and crosses) in common is computed as the intersection between selected parents (crosses) in run 1 and selected parents (crosses) in run 2 divided by the number of crosses selected in both runs.*

| **POPULATION** | **CRITERION** | **CONSTRAINTS** | | | | | |
| --- | --- | --- | --- | --- | --- | --- | --- |
|  |  | **TRUE** | | | **ESTIMATED** | | |
|  |  | **TOP1 parent** | **TOP20 parents** | **TOP100 parents** | **TOP1 parent** | **TOP20 parents** | **TOP100 parents** |
| Unselected | PM | 4%±0 | 76%±0 | 100%±0 | 4%±0 | 76%±0 | 100%±0 |
|  | PROBA | 4%±0 | 71%±4 | 99%±1 | 4%±0 | 70%±3 | 98%±1 |
|  | UC1 | 4%±0 | 69%±4 | 99%±0 | 4%±0 | 73%±3 | 99%±0 |
|  | UC2 | 4%±0 | 55%±5 | 94%±4 | 4%±0 | 63%±6 | 97%±2 |
|  | UC3 | 4%±0 | 69%±4 | 99%±0 | 4%±0 | 65%±5 | 98%±1 |
|  | EMBV | 4%±0 | 69%±4 | 99%±0 | 4%±0 | 73%±3 | 100%±0 |
|  | OHV | 3%±1 | 42%±6 | 84%±5 | 4%±0 | 55%±6 | 92%±5 |
| Selected | PM | 4%±0 | 76%±0 | 100%±0 | 4%±0 | 76%±0 | 100%±0 |
|  | PROBA | 4%±0 | 72%±3 | 99%±0 | 4%±0 | 70%±5 | 99%±0 |
|  | UC1 | 4%±0 | 66%±5 | 99%±0 | 4%±0 | 74%±2 | 99%±0 |
|  | UC2 | 4%±0 | 47%±7 | 88%±5 | 4%±0 | 68%±4 | 99%±0 |
|  | UC3 | 4%±0 | 70%±4 | 98%±0 | 4%±0 | 62%±6 | 98%±1 |
|  | EMBV | 4%±0 | 68%±5 | 99%±0 | 4%±0 | 75%±2 | 100%±0 |
|  | OHV | 3%±1 | 37%±6 | 73%±6 | 4%±0 | 61%±4 | 97%±1 |
|  | | **NO CONSTRAINTS** | | | | | |
|  |  | **TRUE** | | | **ESTIMATED** | | |
|  |  | **TOP1 parent** | **TOP20 parents** | **TOP100 parents** | **TOP1 parent** | **TOP20 parents** | **TOP100 parents** |
| Unselected | PM | 23%±9 | 92%±9 | 100%±0 | 21%±9 | 92%±9 | 100%±0 |
|  | PROBA | 23%±9 | 90%±9 | 100%±0 | 21%±9 | 90%±8 | 100%±0 |
|  | UC1 | 22%±11 | 85%±8 | 100%±0 | 21%±10 | 88%±8 | 100%±0 |
|  | UC2 | 18%±13 | 72%±3 | 97%±0 | 18%±11 | 80%±8 | 98%±0 |
|  | UC3 | 22%±11 | 85%±8 | 100%±0 | 20%±9 | 89%±7 | 100%±0 |
|  | EMBV | 22%±11 | 82%±7 | 100%±0 | 21%±10 | 87%±8 | 100%±0 |
|  | OHV | 12%±11 | 54%±9 | 85%±4 | 16%±12 | 71%±7 | 91%±4 |
| Selected | PM | 25%±8 | 90%±7 | 100%±0 | 25%±9 | 91%±8 | 100%±0 |
|  | PROBA | 25%±8 | 88%±7 | 100%±0 | 26%±9 | 87%±9 | 100%±0 |
|  | UC1 | 22%±10 | 84%±7 | 100%±0 | 26%±10 | 87%±8 | 100%±0 |
|  | UC2 | 14%±11 | 64%±8 | 85%±0 | 25%±11 | 81%±8 | 100%±0 |
|  | UC3 | 22%±10 | 84%±7 | 100%±0 | 25%±9 | 87%±8 | 100%±0 |
|  | EMBV | 21%±10 | 81%±7 | 98%±0 | 26%±10 | 86%±8 | 100%±0 |
|  | OHV | 7%±7 | 48%±11 | 79%±6 | 22%±13 | 75%±6 | 93%±2 |

**Supplementary Table S2: Contribution of parents, focusing on the best parent (Top1), the 10 best parents (Top20) and the best 100 parents (Top100), depending on scenario and criterion.** Parents are ranked based on TBV in TRUE scenario and GEBV in ESTIMATED scenario. The contribution is computed as the number of progenies allocated to a parent, divided by the total possible contributions (*e.g.*, 3300*2). Note that in CONSTRAINTS scenario, the constraints C4 forces the number of progenies per parent to be lower than 250 (*e.g.*, 4% of total progeny 3300 * 2).

| **POPULATION** | **CRITERION** | **CONSTRAINTS** | | | | | |
| --- | --- | --- | --- | --- | --- | --- | --- |
|  |  | **TRUE** | | | **ESTIMATED** | | |
|  |  | **TOP1 cross** | **TOP20 crosses** | **TOP100 crosses** | **TOP1 cross** | **TOP20 crosses** | **TOP100 crosses** |
| Unselected | PM | 0%±1 | 5%±2 | 29%±9 | 0%±1 | 6%±2 | 33%±4 |
|  | PROBA | 2%±0 | 16%±2 | 30%±2 | 2%±0 | 15%±3 | 23%±6 |
|  | UC1 | 1%±1 | 7%±1 | 24%±3 | 0%±0 | 5%±3 | 22%±5 |
|  | UC2 | 1%±1 | 5%±2 | 19%±2 | 0%±0 | 5%±2 | 17%±4 |
|  | UC3 | 2%±0 | 16%±2 | 30%±2 | 2%±0 | 15%±3 | 23%±6 |
|  | EMBV | 1%±1 | 7%±1 | 24%±3 | 0%±0 | 4%±3 | 23%±5 |
|  | OHV | 1%±1 | 2%±2 | 9%±3 | 0%±0 | 5%±3 | 14%±4 |
| Selected | PM | 0%±0 | 7%±3 | 32%±7 | 0%±0 | 6%±2 | 30%±3 |
|  | PROBA | 2%±0 | 15%±4 | 33%±0 | 2%±0 | 15%±2 | 34%±0 |
|  | UC1 | 1%±1 | 6%±2 | 21%±4 | 0%±1 | 5%±2 | 24%±4 |
|  | UC2 | 1%±1 | 3%±3 | 11%±0 | 0%±1 | 4%±3 | 21%±4 |
|  | UC3 | 2%±0 | 15%±4 | 33%±0 | 2%±0 | 15%±2 | 34%±0 |
|  | EMBV | 1%±1 | 5%±2 | 23%±2 | 0%±1 | 5%±3 | 24%±4 |
|  | OHV | 0%±0 | 0%±0 | 1%±0 | 0%±0 | 4%±3 | 18%±2 |
|  | | **NO CONSTRAINTS** | | | | | |
|  |  | **TRUE** | | | **ESTIMATED** | | |
|  |  | **TOP1 cross** | **TOP20 crosses** | **TOP100 crosses** | **TOP1 cross** | **TOP20 crosses** | **TOP100 crosses** |
| Unselected | PM | 2%±0 | 36%±0 | 100%±0 | 2%±0 | 36%±0 | 100%±0 |
|  | PROBA | 2%±0 | 36%±1 | 98%±0 | 2%±0 | 35%±2 | 95%±0 |
|  | UC1 | 2%±0 | 32%±3 | 85%±3 | 2%±0 | 34%±4 | 85%±10 |
|  | UC2 | 2%±0 | 19%±4 | 49%±8 | 2%±0 | 24%±7 | 50%±18 |
|  | UC3 | 2%±0 | 32%±3 | 85%±7 | 2%±0 | 34%±4 | 81%±11 |
|  | EMBV | 2%±0 | 30%±3 | 78%±4 | 2%±0 | 33%±5 | 78%±13 |
|  | OHV | 2%±0 | 10%±4 | 24%±13 | 2%±0 | 19%±6 | 37%±15 |
| Selected | PM | 2%±0 | 36%±0 | 100%±0 | 2%±0 | 36%±0 | 100%±0 |
|  | PROBA | 2%±0 | 36%±1 | 97%±3 | 2%±0 | 36%±1 | 98%±3 |
|  | UC1 | 2%±0 | 31%±4 | 72%±7 | 2%±0 | 35%±2 | 97%±1 |
|  | UC2 | 2%±0 | 10%±5 | 37%±5 | 2%±0 | 29%±4 | 80%±3 |
|  | UC3 | 2%±0 | 30%±3 | 72%±9 | 2%±0 | 35%±2 | 96%±0 |
|  | EMBV | 2%±0 | 27%±4 | 66%±8 | 2%±0 | 35%±2 | 96%±4 |
|  | OHV | 2%±0 | 4%±0 | 8%±1 | 2%±0 | 21%±6 | 58%±2 |

**Supplementary Table S3: Proportion of progeny allocated to the best cross (Top1), to the 20 best crosses (Top20) and to the best 100 crosses (Top100), depending on scenario and criterion.** Crosses are ranked based on average TBV in TRUE scenario and average GEBV in ESTIMATED scenario. Note that in CONSTRAINTS scenario, the constraint C2 forces the number of progenies per cross to range between 5 and 60 (*e.g.*, 2% of total progeny 3300) and the constraint C3 forces the number of crosses to select to range between 200 and 300. In NO CONSTRAINT scenario, the 55 best crosses for each criterion were selected and received 60 progenies (total progeny 3300).
